# Acetyl-CoA synthase 1 mediates metabolic reprogramming to promote proliferation and metastasis of osteosarcoma^☆^^☆☆^

**DOI:** 10.1016/j.jot.2026.101052

**Published:** 2026-04-01

**Authors:** Xiuming He, Mengliang Luo, Chenyang Wang, Lingkai Kong, Jianglong Li, Jianye Tan, Junhao Chen, Yi Nie, Hantao Chen, Zhipeng Zou, Zexin Su, Lijun Lin

**Affiliations:** a Department of Joint and Orthopedics, Zhujiang Hospital, Southern Medical University, Guangzhou, 510280, PR China; b Department of Orthopedics, The Second Affiliated Hospital of Chongqing Medical University, Chongqing Medical University, Chongqing, 400072, PR China; c Department of Gerontology, Zhujiang Hospital, Southern Medical University, Guangzhou, 510280, PR China; d Department of Orthopedics, Zhongshan Torch Development Zone People's Hospital, Zhongshan, 528400, PR China; e Guangdong Provincial Key Laboratory of Bone and Joint Degeneration Diseases, Department of Cell Biology, School of Basic Medical Sciences, Southern Medical University, Guangzhou, 510515, PR China

**Keywords:** Osteosarcoma, ACSS1, SCD, Mitochondria, Lipid metabolism

## Abstract

**Background:**

Although the combination of surgery and chemotherapy has dramatically improved the prognosis of patients with osteosarcoma (OS), the prognosis of OS patients with metastatic or recurrent tumors remains poor. Here, we found significant upregulation of the acetyl-CoA synthesis-related pathway in OS patients with metastases.

**Methods:**

Bioinformatics analysis identified ACSS1 as a key metastasis-related gene in OS, with clinical OS specimens validating its association with poor prognosis. In vitro, ACSS1 knockdown in 143B and SJSA1 cells was used to assess proliferation (EdU, colony formation), migration/invasion (Transwell, 3D spheroids), and mitochondrial function (Seahorse, TEM). Untargeted metabolomics and transcriptomics characterized ACSS1-regulated lipid pathways, while *in vivo* validation employed nude mouse models of orthotopic xenograft and tail vein metastasis.

**Results:**

Notably, the expression of Acetyl-coenzyme A synthetase 1 (ACSS1), an enzyme critical for acetyl-CoA synthesis, was negatively correlated with pulmonary metastasis-free and overall survival in OS patients. ACSS1 depletion decreases the metastatic ability and energy metabolism of OS cells, resulting in reactive oxygen species (ROS) accumulation. Mechanistically, ACSS1 exerts acetyl-CoA synthetase activity and maintains dynamic homeostasis of acetyl-CoA metabolism in the mitochondria. It promotes lipid synthesis and regulates metabolic homeostasis by inducing stearoyl coenzyme A desaturase (SCD) expression, which can fuel the metastasis of OS cells and promotes their survival.

**Conclusion:**

ACSS1 drives OS metastasis by maintaining mitochondrial acetyl-CoA homeostasis and activating SCD-dependent lipid synthesis. Targeting the ACSS1/SCD axis offers a promising strategy for metastatic OS.

**The translational potential of this article:**

This study reveals the therapeutic potential of targeting ACSS1/SCD axis, for example, by specific small-molecule inhibitors in treating osteosarcoma metastasis, in addition to conventional chemotherapies.

## Graphical abstract

**Figure:**
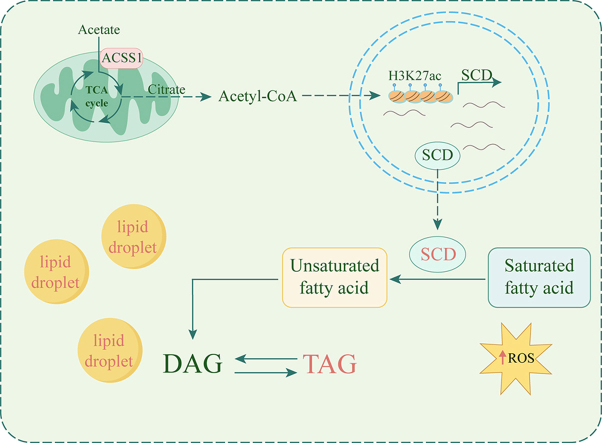

## 1 Introduction

Metastasis is the leading cause of mortality in most cancers and is responsible for 90% of all cancer-related deaths [1]. Lung metastasis is the most common form of cancer in osteosarcoma (OS) and is significantly associated with poor prognosis [2,3]. Despite advances in neoadjuvant chemotherapy and surgical methods for treating OS, the five-year survival of patients with metastatic disease remains poor, once the patient has metastasis or treatment fails, the overall survival rate is going to drop significantly to 20-30% [[4], [5], [6]]. Additionally, more than 30% of patients have metastases at initial diagnosis. Hence, further studies are necessary to unravel the mechanisms that drive OS metastasis and develop novel therapeutic strategies for treating OS.

Metabolic reprogramming of cancer has been described as a hallmark of human malignancies [7,8]. Unlike other tumors, hypoxic tumor microenvironments, characteristic of sarcomas, modify metabolism and correlate with worse prognosis. However, Detailed data regarding OS metabolome is relatively sparse. Reprogramming of lipid metabolism is the most prominent feature of some tumors [9,10]. Notably, lipid metabolic disorders facilitate the rapid proliferation and metastasis of tumor cells and promote aberrant communication between cancer and stromal cells [11]. Recent studies have disclosed that de novo fatty acid synthesis and β-oxidation increase lipid storage, cancer cell proliferation, and metastasis [12]. Therefore, targeting tumor lipid metabolism has been considered a new therapeutic strategy. Under hypoxic conditions, OS may experience a transition from amino acid and carbohydrate consumption to lipid consumption throughout the entire lung metastasis period. Increased levels of metabolites linked with lipid metabolism and amino acid biosynthesis pathways are characteristic of OS. The recognition of these metabolic features lays the foundation for the study of osteosarcoma, which helps with primary diagnosis and metastasis prediction, and enables better disease follow-up in the near future.

Acetyl-CoA, a two-carbon end product of glucose, fatty acid, and amino acid catabolism, functions both as a central metabolic intermediate and as a key signaling nexus [13]. It is a feedstock for fatty acid synthesis and influences the activities of several fatty acid synthases [14]. Accordingly, acetyl-CoA plays an important role in lipid metabolism. In mammals, acetyl-CoA is produced by three enzymes: ATP-citrate lyase (ACLY), pyruvate dehydrogenase complex (PDHc), and acetyl-CoA synthetase (ACSS) [15]. ACLY is a cytosolic enzyme that catalyzes citrate to acetyl-CoA and oxaloacetate [16]. PDHc, an enzyme complex that catalyzes pyruvate conversion into acetyl-CoA, interconnects glycolysis with the tricarboxylic acid cycle and lipogenesis [17]. The acetyl-CoA synthase family (ACSS1, ACSS2, and ACSS3) are key enzymes involved in activating acetate in acetyl-CoA [18].

Studies have shown that ACSS1-mediated acetyl coenzyme metabolism is essential for tumor development [[19], [20], [21], [22], [23]]. ACSS1 promotes EMT transformation in bladder cancer cells and predicts radiation-induced breast cancer recurrence [21]. Global gene expression profiling from hepatocellular carcinoma revealed that the metabolic pattern of mitochondrial acetate-promoting fatty acid biosynthesis by upregulating ACSS1 is an important feature of metabolic dysregulation in hepatocellular carcinoma. ACSS1 is significantly associated with tumor growth and malignant progression under hypoxic conditions in hepatocellular carcinoma cells [20]. In vivo mouse studies have revealed that alterations in ACSS1 activity are linked to the dysregulation of lipid metabolism and processes of cellular aging, these observations suggest that ACSS1 potentially plays a crucial role in the intricate regulation of both lipid homeostasis and cellular function [24]. However, the role of ACSS1 in OS, a hard-tissue sarcoma exhibiting distinct metabolic phenotypes from these cancer types originated from epithelial tissues, is unclear.

In the current study, we found that ACSS1 expression was significantly higher in OS samples with metastasis than those without. Meanwhile, ACSS1 expression was significantly associated with pulmonary metastasis-free and overall survival. ACSS1 depletion reduces the invasion and migration of OS cells and inhibits tumor growth *in vivo*. Mechanistically, ACSS1 not only stabilizes mitochondrial energy metabolism, but also promotes fatty acid synthesis and prevents lipid peroxidation by activating stearoyl coenzyme A desaturase (SCD). Our results demonstrated that ACSS1 can affect acetyl-CoA levels to control OS metabolism. This study provides new targets for tumor metabolic therapy.

## 2 Results

### 2.1 ACSS1 is related to OS metastasis

We performed a bioinformatic analysis to assess differences in gene expression in patients with OS with metastases or without (GSE21257, named thereafter the discovery cohort). Gene Ontology (GO) enrichment analysis revealed significant correlations between mitochondrial composition and energy metabolic pathways in patients with metastatic OS (Fig. 1A–B). Particularly, the biological synthesis pathway of acetyl-CoA, which is central to mitochondrial energy metabolism, was significantly upregulated in patients with OS who developed metastases (Fig. 1C). Therefore, by further screening the key genes (ACSS1, ACSS2, ACLY, PDHA, and PDHB) of acetyl-CoA synthesis (Fig. 1D), we found that high ACSS1 expression was associated with poor pulmonary metastasis-free and overall survival (Fig. 1E and S1A). Next, we collected OS specimens to verify the results obtained from the discovery cohort. Immunohistochemical examination confirmed that ACSS1 expression was significantly higher in OS tissues with metastases than in those without (Fig. 1F). Immunofluorescence staining experiment suggested that ACSS1 was mainly localized to the mitochondria (Fig. 1G). Additionally, western blotting assay showed that ACSS1 expression was higher than that of human mesenchymal stem cell (HMSC) in almost all OS cell lines (Fig. 1H).

**Fig. 1:**
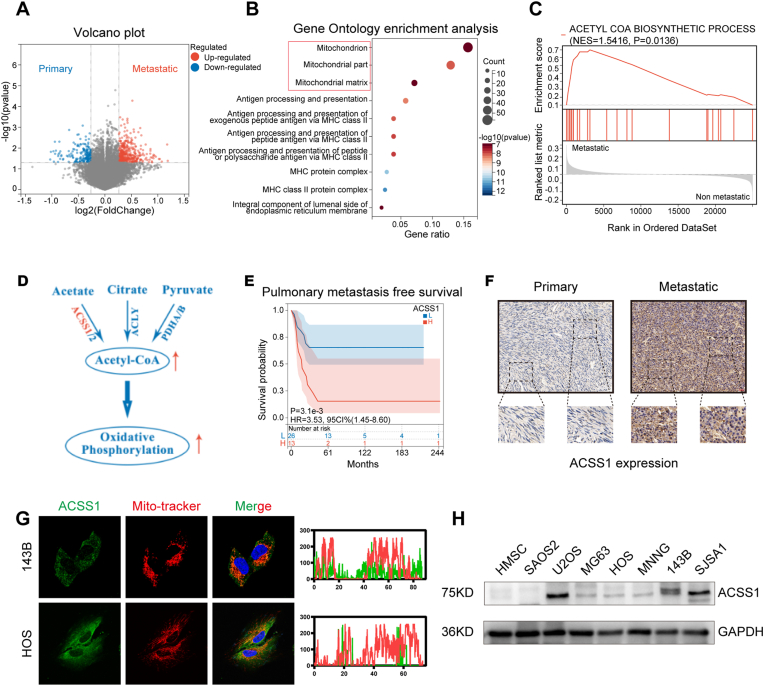
**A** Volcano plots showing the differential genes in metastatic versus non-metastatic OS tissues in GSE21257. **B** GO functional enrichment analysis of significantly differential genes. **C** The acetyl coenzyme A biosynthetic process in GSEA analysis. **D** Pattern diagram of acetyl coenzyme A biosynthetic process. **E** Pulmonary metastasis-free survival analysis of different ACSS1 expression in OS patients. **F** Immunohistochemical analysis of ACSS1 expression in specimens from patients with OS. **G** Immunofluorescence analysis showing the localization of ACSS1 in OS cells. **H** Protein expression of ACSS1 in OS cell lines and HMSC.

### 2.2 ACSS1 promotes OS proliferation and metastasis

To validate the biological functions of ACSS1 in OS, 143B and SJSA1 cell lines were used in further experiments. The ACSS1 depletion was confirmed using Western blotting in the above two cell lines (Fig. 2A). Next, ACSS1 knockdown inhibited OS cell proliferation, as observed by EdU doping and clone formation assay (Fig. 2B–C, Fig. S1B). Additionally, ACSS1 knockdown repressed the migration and invasion of OS cells, as verified by transwell and three-dimensional (3D) spheroid formation assays (Fig. 2D–I, S1G, and S1H). Similar results were obtained in the scratch healing assays (Fig. S1C–F). Epithelial-mesenchymal transition (EMT) and microfilament growth positively correlate with tumor metastasis [25]. Therefore, the expressions of vimentin, E-cadherin, and N-cadherin, three important hallmarks of EMT, were examined by western blotting. The results showed that a significant reduction in EMT occurred in ACSS1-depleted OS cells (Fig. 2J). In addition, ACSS1 knockdown inhibited microfilament growth, as evidenced by F-actin staining (Fig. 2K and L). These data demonstrate that ACSS1 is crucial for OS proliferation, migration, and invasion.

**Fig. 2:**
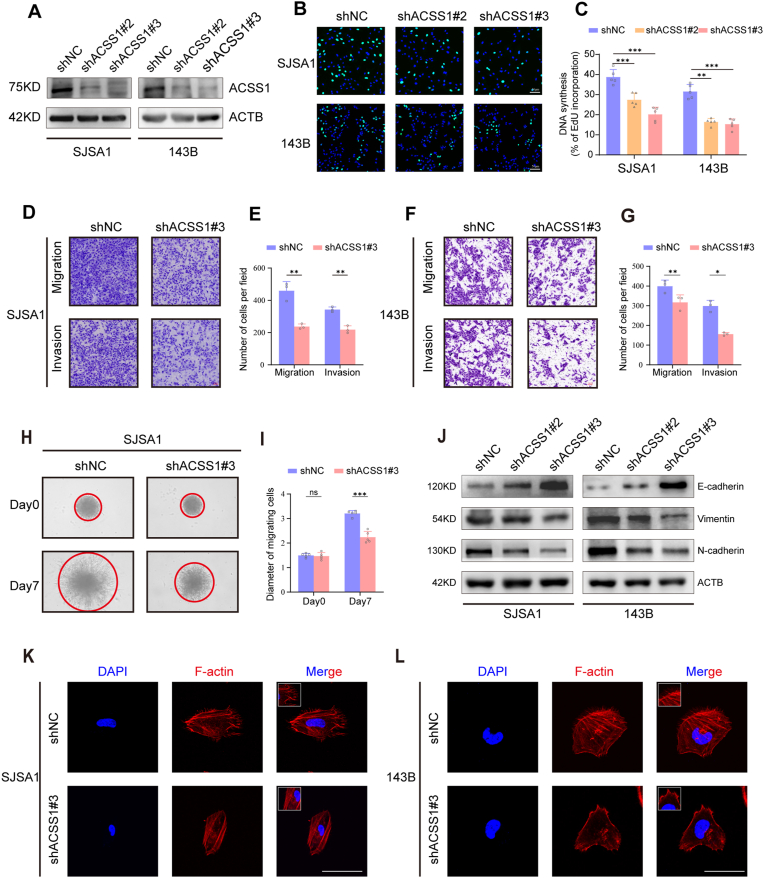
**A** Western blotting detection of the protein knockdown efficiency of ACSS1 in SJSA1 and 143B cells. **B** EdU doping assay showing the DNA replication capacity of SJSA1 and 143B cells. **C** For the statistical graph in b, five random captures were taken for statistical purposes. **D-E** Migration and invasion ability of SJSA1 cells detected using Transwell assay. **F-G** Migration and invasion ability of 143B cells detected using Transwell assay. **H** 3D sphere-forming assay showing the invasive ability of SJSA1 cells. **I** The statistics are plotted in terms of the diameter of the infiltrating cells based on h. **J** Western blotting was used to detect vimentin, N-cadherin, and E-cadherin protein expression levels. **K-I** Phalloidin staining detecting the number of pseudopods on the surface of OS cells after ACSS1 knockdown. Scale bar: 100 μm. Data are expressed as the mean ± SD. ∗*p* < 0.05; ∗∗*p* < 0.01; ∗∗∗*p* < 0.001; ns. not significant.

### 2.3 ACSS1 maintains dynamic mitochondrial homeostasis

In OS patients with metastasis, high ACSS1 expression is associated with significantly altered mitochondria-related pathways. Thus, we hypothesized that the metastatic ability of OS may be related to mitochondrial function and energy production. To test this hypothesis, we examined the effects of ACSS1 depletion on the oxygen consumption rate (OCR) of cells. The results indicated that ACSS1 knockdown significantly reduced OCR levels compared to control cells, suggesting that mitochondrial function was significantly inhibited (Fig. 3A–B). Furthermore, JC-1 fluorescence staining revealed decreased mitochondrial membrane potential (ΔΨm) accompanied by ACSS1 expression, validating the above results (Fig. 3F and G, S2A, and S2B). In addition, transmission electron microscopy (TEM) was used to observe the effect of ACSS1 depletion on mitochondrial structure in SJSA1 and 143B cells. Most of the mitochondria bulged, broken, and cristae fractured in the knockdown group of ACSS1 (Fig. 3C–D). The majority of intracellular reactive oxygen species (ROS) originates from mitochondria. ACSS1 knockdown resulted in a marked increase in ROS (Fig. 3E and S2C), suggesting that ACSS1 is important for maintaining mitochondrial function and structure in OS cells. Furthermore, ACSS1 knockdown led to a statistically significant decrease in overall acetyl-CoA levels in both cell lines (Fig. 3H–I).

**Fig. 3:**
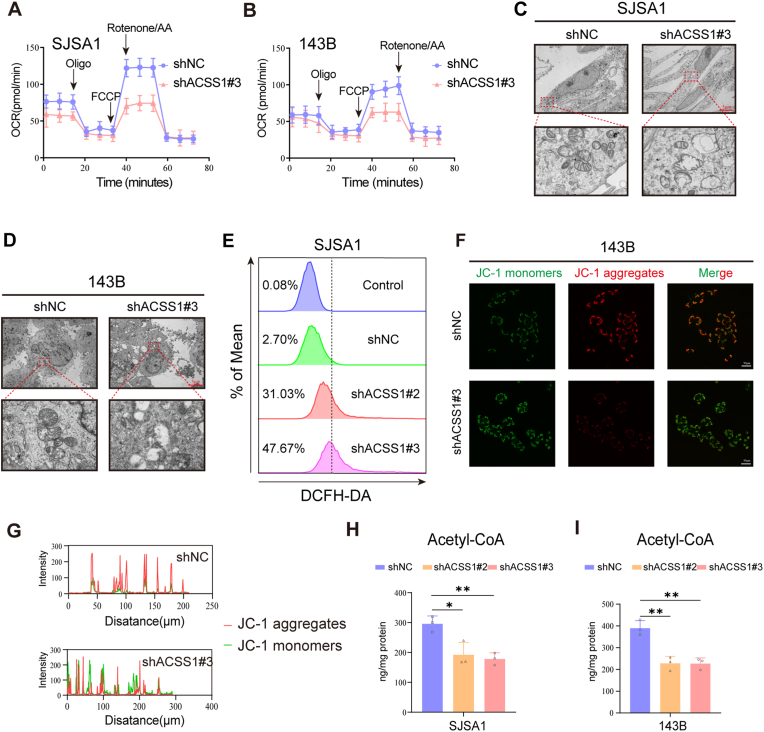
**A-B** Seahorse assay to detect intracellular oxygen consumption and mitochondrial reserve capacity in OS cells after ACSS1 knockdown. **C-D** Transmission electron microscopy of mitochondrial structure in OS cells after reduced ACSS1 expression. **E** Intracellular reactive oxygen species levels in SJSA1 cells under ACSS1 knockdown were detected using flow cytometry. **F-G** Staining of mitochondrial JC-1 membrane potential and fluorescence intensity graphs in 143B cells after ACSS1 knockdown. **H-I** Alterations in acetyl coenzyme A in OS cells after ACSS1 knockdown were detected using ELISA. Data are expressed as the mean ± SD. ∗*p* < 0.05; ∗∗*p* < 0.01; ∗∗∗*p* < 0.001; ns. not significant.

### 2.4 ACSS1 depletion significantly altered lipid homeostasis in OS cells

ACSS1 is a key enzyme in acetyl-CoA synthesis. To evaluate the effect of ACSS1 depletion on OS metabolism, the metabolic pathways affected by ACSS1 depletion and consequent reduction of acetyl-CoA was explored using untargeted metabolomics. The results showed that lipid and lipid-like molecules were most altered (Fig. S3a–b). According to the enrichment results of differential metabolites, ACSS1 knockdown mainly affected glycerophospholipid and fatty acid metabolism pathways, especially the reduction in palmitic acid levels (Fig. 4A and C). By integrating the metabolomics with transcriptomic data, we focused on fatty acid biosynthesis, degradation, and unsaturated fatty acid biosynthesis pathways (Fig. 4B–S2D, and S2E).Consistent with palmitate reduction, triglyceride (TG) synthesis using fatty acids (FA) as substrates was significantly reduced in ACSS1-depleted cells (Fig. 4D). Consistently, BODIPY493/503 staining demonstrated that reduced ACSS1 expression resulted in reduced lipid droplet storage (Fig. 4E and F, S3D, and S3E). Untargeted metabolomics additionally revealed significant reduction of some structural lipids in ACSS1-depleted cells, such as phosphatidylinositol (PI). In contrast, lysophosphatidylcholine (LPC) and lysophosphatidylserine (LPS) were elevated (Fig. 4H–J). Protein expression of key genes regarding monounsaturated fat synthesis (MUFA), such as stearoyl coenzyme A desaturase (SCD) and fatty acid desaturase 2 (FADS2), was reduced (Fig. 4G and S3C). BODIPY581/591 staining showed that lipid-derived ROS production was significantly elevated in ACSS1 knockdown cells (Fig. 5A). In conclusion, these data further suggested that ACSS1 maintains lipid homeostasis to prevent OS cells from oxidative stress, enhancing the proliferation and metastatic ability of them.

**Fig. 4:**
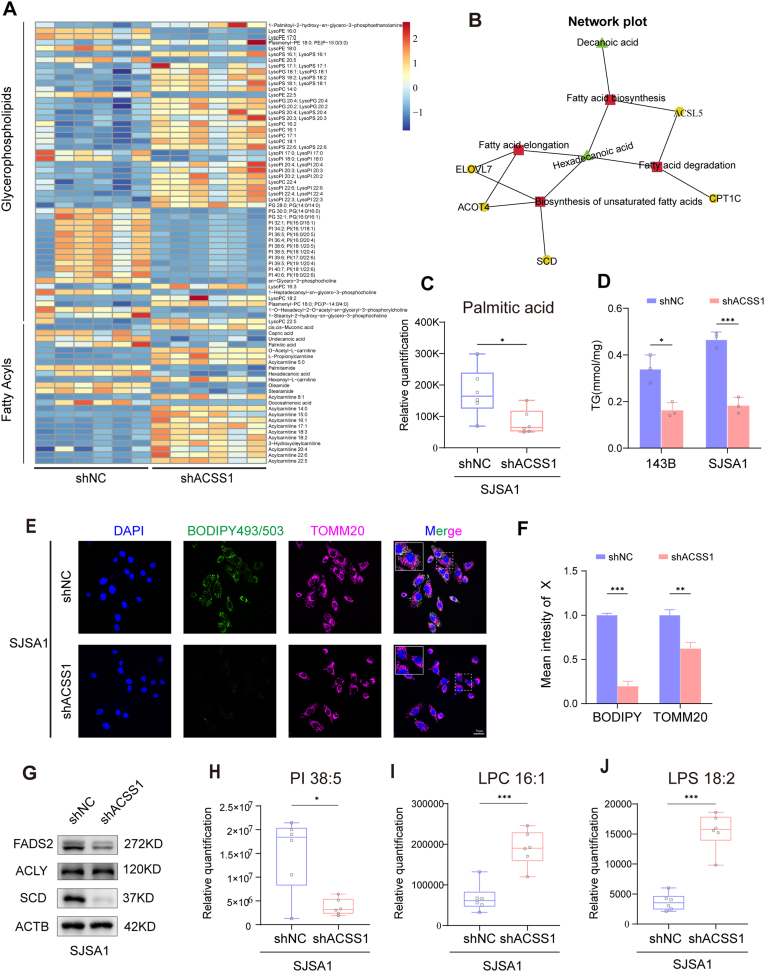
**A** Heatmap of representative metabolite species in ACSS1-knockdown SJSA1 cells (n = 6) versus control cells (n = 6) analyzed using untargeted metabolomics. **B** A net plot of the combined transcriptomics and untargeted metabolomic analysis of ACSS1 knockdown in SJSA1 cells. **C** Palmitate metabolite content in untargeted metabolomics after ACSS1 knockdown. **D** Determination of TG content after ACSS1 knockdown. **E-F** Detection of lipid droplet content and TOMM20 expression by immunofluorescence in SJSA1 cells. **G** Western blotting detection of fatty acid metabolism-related gene changes in SJSA1 cells. **H–J** Expression of PI, LPC, and LPS in untargeted metabolomics. Data are expressed as the mean ± SD. ∗*p* < 0.05; ∗∗*p* < 0.01; ∗∗∗*p* < 0.001; ns. not significant.

**Fig. 5:**
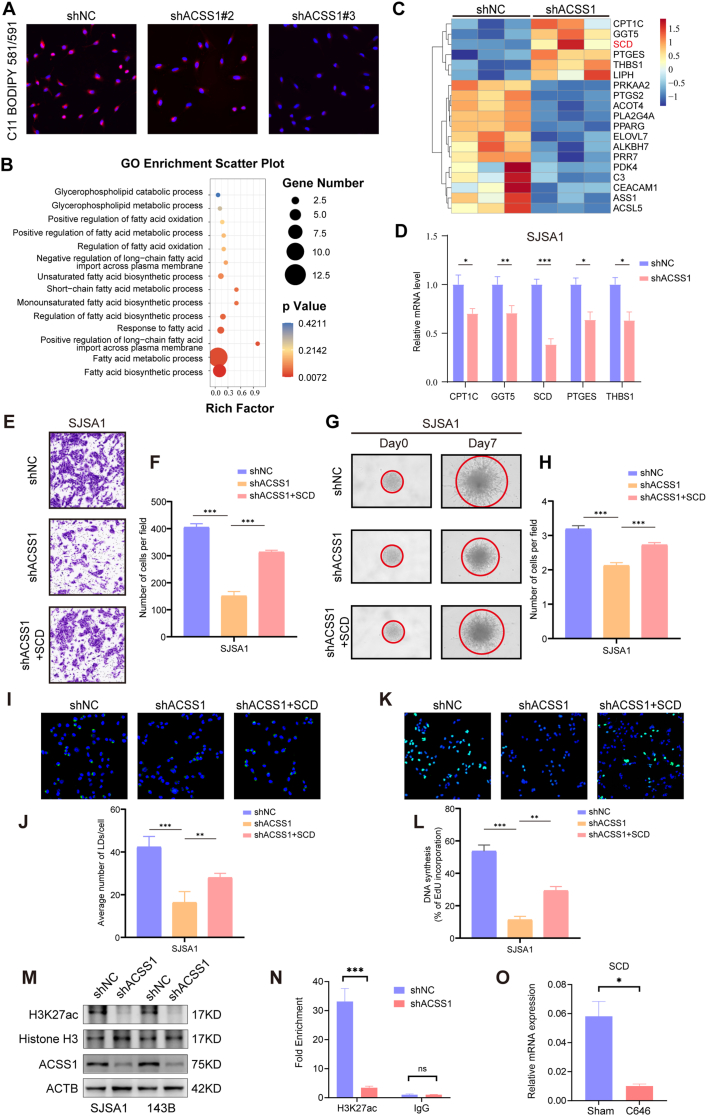
**A** C11 BODIPY581/591 assay for lipid peroxidation levels after ACSS1 knockdown in SJSA1 cells. **B** Heatmap of transcriptomics of knockdown ACSS1 (n = 3). **C** GO analysis was conducted to look for biological processes after ACSS1 knockdown. **D** Relative mRNA expression of the five candidate downstream genes was determined using qRT-PCR. **E-F** Transwell assay to detect the effect of ACSS1/SCD axis on OS cell invasion. **G-H** 3D sphere-forming assay to detect the effect of the ACSS1/SCD axis on cell tumorigenesis. **I-J** Effect of ACSS1/SCD axis on lipid droplet content detected by BODIPY 493/503 staining. **K-L** EdU doping assay to detect the effect of the ACSS1/SCD axis on cell proliferation. **M** Western blotting detection of acetylation H3K27 in osteosarcoma cells. **N** ChIP‐qPCR analysis showing reduced H3K27ac enrichment after ACSS1 knockdown. **O** Relative mRNA expression of SCD after inhibition of P300 with C646. Data are expressed as the mean ± SD. ∗*p* < 0.05; ∗∗*p* < 0.01; ∗∗∗*p* < 0.001; ns. not significant.

### 2.5 ACSS1 remodels fatty acid synthesis of OS via SCD

To validate the critical role of SCD downstream of ACSS1, we performed a clustering analysis of transcriptomics and screened the main downstream gene SCD affected by ACSS1 using qRT-PCR (Fig. 5B–D). To confirm that ACSS1 remodels fatty acid synthesis and promotes OS metastasis via SCD, we overexpressed SCD in ACSS1-knockdown osteosarcoma cells for subsequent rescue experiments. Transwell and 3D sphere-forming experiments showed that overexpression of SCD rescued the impaired cell invasion capability induced by ACSS1 knockdown (Fig 5E–H). Moreover, it led to an increase in lipid droplet content (Fig. 5I–J). Meanwhile, EdU doping assays revealed that the proliferation ability of OS cells was similarly increased when SCD was overexpressed (Fig. 5K–L). Conversely, we overexpressed ACSS1 in SAOS2 cell lines and used the SCD inhibitor Cay to confirm the ACSS1-SCD pathway. As expected, ACSS1 overexpression promoted SCD expression (Fig. s4A). However, while ACSS1 overexpression could promote the ability of OS cells to migrate and invade, SCD inhibition by Cay reversed these phenotypes (Fig. S4bB-E). Additionally, proliferation of OS cells was similarly reduced when SCD expression was inhibited (Fig. s4H-I). Furthermore, lipid droplet accumulation induced by ACSS1 overexpression was significantly suppressed by Cay treatment(Fig. s4F-G).

Taken together, these results suggest that ACSS1 remodels lipid metabolism of OS cells to enhance their proliferation, migration, and invasion via SCD.

### 2.6 ACSS1 activates H3K27 acetylation of *SCD* promoter

Since ACSS1 increases the synthesis of acetyl-CoA, we hypothesized that this excess of acetyl-CoA will enter the nucleus and promote the promoter acetylation of target genes including *SCD* and activate their transcription. As expected, ACSS1 depletion reduced the acetylation H3K27 in osteosarcoma cells (Fig. 5M), resulting in a significant decrease in H3K27AC occupancy at *SCD* promoter (Fig. 5N). Meanwhile, the use of histone acetyltransferase p300 inhibitor C646 can also suppress the expression level of SCD mRNA (Fig. 5O).

### 2.7 ACSS1 promotes OS proliferation and metastasis *in vivo*

**Fig. 6:**
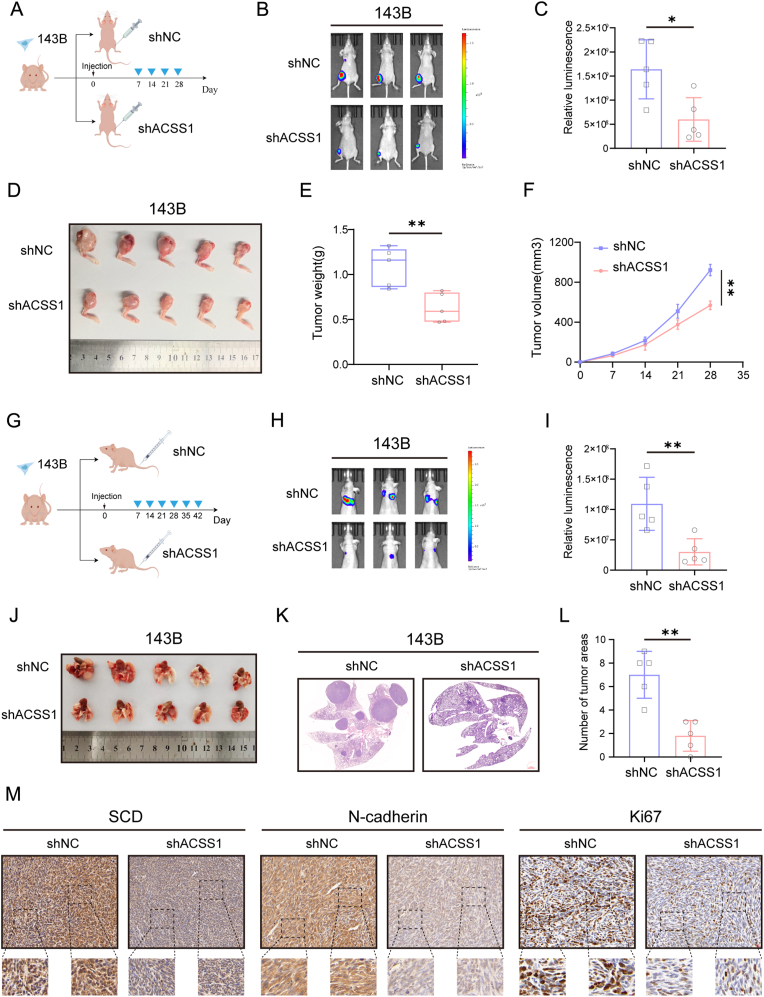
**A** Diagram of the orthotopic xenograft model of the right tibia in nude mice (n = 5). **B-C** A small animal *in vivo* optical imaging system was used to observe the fluorescence intensity values between knockdown ACSS1 and controls. **D–F** Tumor bulk, weight, and volume between the knockdown ACSS1 and control groups. **G** Diagram of the nude mouse tail vein lung metastasis xenograft model (n = 5). **H-I** The effects of the ACSS1 knockdown on lung metastasis formation were visualized by live imaging in small animals. **J** A gross view of the metastases in the lungs. **K-L** H&E staining was utilized to assess the pathological findings in the lungs. **M** Immunohistochemical staining was performed to observe the effect of ACSS1 on the expression of SCD, N-cadherin, and Ki67. Data are expressed as the mean ± SD. ∗*p* < 0.05; ∗∗*p* < 0.01; ∗∗∗*p* < 0.001; ns. not significant.

Next, we constructed an *in vivo* xenograft study using orthotopic xenograft models (Fig. 6A). Tumor growth was observed by *in vivo* live imaging, and the results showed that ACSS1 knockdown inhibited tumor growth (Fig. 6B–C, 6F). Subsequently, tumor tissues were removed and analyzed using immunohistochemical staining. The results revealed that reduced ACSS1 expression inhibited the expression of Ki67, an indicator of tumor growth and proliferation (Fig. 6D, E, and M), and inhibited the occurrence of EMT (Fig. 6M–S3F). Furthermore, tail vein injection of OS cells was used to verify the effect of ACSS1 expression on OS cell metastasis *in vivo* (Fig. 6G). The results showed that the ACSS1 knockdown reduced OS metastatic ability (Fig. 6H–L). In addition, along with the decrease in ACSS1 expression, the expression of SCD in OS tissue also significantly decreased (Fig. 6M). In summary, ACSS1 is essential for the proliferation and metastatic ability of OS.

### 2.8 ACSS1 expression is positively correlated with SCD in OS metastasis and linked to poor survival

**Fig. 7:**
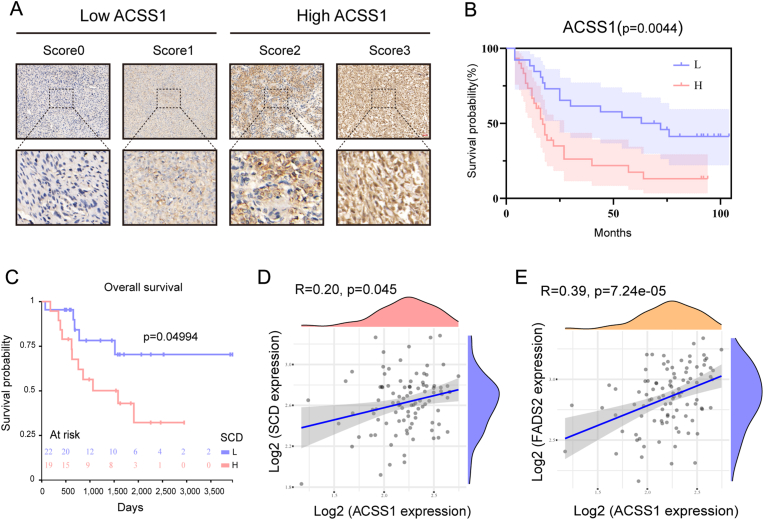
**A** Immunohistochemical scores distinguish high and low ACSS1 expression in tissue samples. **B** Analysis of ACSS1 expression in tissues and overall survival of OS patients. **C** Prognostic analysis of SCD and overall survival in patients with OS. **D** Correlation analysis between ACSS1 and SCD. **E** Correlation analysis between ACSS1 and FADS2.

To investigate the clinical value of ACSS1, we performed immunohistochemical staining of tissues collected from 56 patients with OS (Fig. 7A–B; Table 1). The results displayed that most patients with high ACSS1 expression developed distant metastases. The prognosis of patients with high ACSS1 expression is poor (Fig. S5). Additionally, correlation analysis and K-M analysis in the Target-OS database showed that ACSS1 positively correlated with fatty acid metabolism-related genes, SCD, and FADS2 (Fig. 7D and E). Furthermore, patients with high SCD expression had poorer overall survival (Fig. 7C). These results demonstrated that ACSS1-SCD axis is critical for OS metastasis and linked with poor survival.

**Table 1:** Clinical characteristics of 56 patients with OS.

Clinicopathological features	Relative ACSS1 expression		*P*-value (χ^2^ test)
	Low(n=26)	High(n=30)	
*Gender*			0.333
male	18	17	
female	8	13	
*Age*			0.136
≤18	4	11	
>18	22	19	
*Distant metastasis*			0.035∗
*Absent*	16	10	
*Present*	10	20	

## 3 Discussion

OS is the most common primary malignant tumor of bone worldwide, with a bimodal incidence pattern that peaks at 18 and 60 years of age [26]. In spite ofsubstantial breakthroughs in treating OS after tumor resection, e.g., neoadjuvant chemotherapy and multiagent induction chemotherapy [27], for patients with metastases, the five-year survival rate i8s still less than 25%. Thus, prevention of OS metastases is of urgent need. The continuous development of metabolomic technology to explore tumor-specific metabolic pathways offers us a better understanding of the mechanisms underlying tumor-specific metastasis [28,29]. In this study, we unravelled the effect ACSS1-SCD on OS. The specific function of ACSS1-SCD was further interrogated by loss-of-function studies in OS cells, xenograft OS models, and a large clinical cohort with 54 OS patients. Targeting fatty acid metabolism mediated by ACSS1-SCD may be promising for the treatment of OS metastasis.

Tumor cell proliferation and metastasis are inseparable from metabolic reprogramming, while mitochondrial homeostasis is critical for cellular energy demand and metabolic homeostasis [30,31]. It has been reported that AHA1 stabilizes IDH1 expression and upregulates metabolic activity to meet the bioenergetic requirements of OS [32]. In this study, ACSS1 was found to maintain mitochondrial structure and function and prevent abnormally elevated levels of oxidative stress. Furthermore, in another study on hepatocellular carcinoma, ACSS1/2-mediated acetic acid metabolism promoted lipid synthesis and tumor growth in HepG2 cells via epistatic modulation of histone acetylation modification [22].Additionally, lipids are essential for cell membrane synthesis and key signaling, and either increased de novo synthesis of FA or inhibition of FAO can promote the malignant biological behavior of cancer cells [33,34]. Previous studies have shown that enzymes associated with the de novo synthesis of FA show a high expression level in many cancers [35,36]. For example, FASN is abnormally upregulated in cervical cancer and promotes lymph node metastasis via cholesterol reprogramming and lymphangiogenesis [36]. Inhibitors of FANS have been applied in the preclinical treatment of hepatocellular carcinoma [37,38]. Moreover, ACC1 is highly expressed in lung and breast cancers and can promote cell proliferation [39,40].

As another key enzyme in de novo lipogenesis, SCD is also considered to play an important role in mediating metastasis in several cancers [41]. SCD generates monounsaturated fatty acids (MUFAs) which contribute to cell growth, survival, differentiation, metabolic regulation and signal transduction [42]. It is reported that growth and metastasis of melanoma was severely restricted by a rationally designed combination therapy of a SCD inhibitor with an isocaloric low-oleic acid diet [43]. In the research of gastric cancer, Ubiquitin-specific protease 7 (USP7) is found to have potential for GC treatment by inducing ferroptosis through SCD regulation, finally prevents tumor migration and invasion [44]. Our investigation underscores the importance of SCD-regulated lipid metabolic homeostasis in facilitating the progression of osteosarcoma. Intriguingly, the regulation of this lipid metabolic equilibrium is itself subject to the influence of ACSS1. Since the SCD inhibitor Aramchol is currently being tested in phase III clinical trials for nonalcoholic steatohepatitis, trial use of this inhibitor to disturb lipid homeostasis in metastatic OS is warranted.

There was no documented role and mechanism of ACSS1 in OS development and progression before. In summary, this study found that ACSS1 expression was upregulated in OS and correlated with a poor prognosis. ACSS1 exerts acetyl-CoA synthetase activity and maintains dynamic homeostasis of acetyl-CoA metabolism in the mitochondria. It promotes lipid synthesis and regulates metabolic homeostasis by modulating SCD expression, providing an adequate energy source for the metastatic progression of OS cells. Additionally, ACSS1 enhances cell proliferation, migration, and invasion, thereby promoting the malignant progression of OS.

## 4 Materials and methods

### 4.1 Cell lines and reagents

The human OS cell lines SAOS2, U2OS, MG63, HOS, MNNG, 143B, and SJSA1 were cultured in Dulbecco's modified Eagle's medium (DMEM) containing 10% fetal bovine serum (FBS), 1% penicillin, and streptomycin. Human bone marrow mesenchymal stem cells (HMSC) were derived from ATCC (Manassas). The cells were cultured in a constant-temperature incubator at 37 °C and 5% CO_2_ with regular fluid exchange procedures. Cay10566 (Cay) was purchased from MedChemExpress (MCE, USA).

### 4.2 Short hairpin RNA (shRNA) and overexpression assay

The shRNA sequences targeting ACSS1 or non-targeting controls were inserted into the LV2-pGLV-U6/puro lentiviral vector (GenePharma, China). Overexpression lentivirus for ACSS1 and paired control vector were provided by GenePharma. Transfection was performed according to the manufacturer's protocol. Stable overexpressed or knockdown OS cell lines were screened and obtained with 1 μg/mL puromycin. Then, transfection efficiency was verified using Western blotting.

### 4.3 RNA sequencing

Briefly, total RNA was extracted from OS cell samples, and the amount and purity of total RNA were quality-controlled using a NanoDrop ND-1000 (NanoDrop, Wilmington, DE, USA). RNA integrity was assessed using Bioanalyzer 2100 (Agilent, CA, USA). Next, bipartite sequencing was performed using an Illumina NovaseqTM 6000 (LC Bio-Technology, Hangzhou, China) according to standard procedures. Differentially expressed genes (DEGs) were defined as genes with a *p*-value <0.05 and an absolute log2 (fold change) > 1.

### 4.4 Untargeted metabolomics analysis

The cells were scraped from the culture dish, centrifuged, and placed in liquid nitrogen for 1 h. Six replicate samples were required for each group, and metabolomic analysis was performed using ultra-performance liquid chromatography-tandem mass spectrometry (UPLC-MS/MS). All samples were acquired by the LC-MS system followed machine orders. Firstly, all chromatographic separations were performed using an UltiMate 3000 UPLC System (Thermo Fisher Scientific, Bremen, Germany). An ACQUITY UPLC T3 column (100 mm∗2.1 mm, 1.8 μm, Waters, Milford, USA) was used for the reversed phase separation. A high-resolution tandem mass spectrometer TripleTOF 6600 (SCIEX, Framingham, MA, USA) was used to detect metabolites eluted form the column. The Q-TOF was operated in both positive and negative ion modes. The curtain gas was set 30 PSI, Ion source gas1 was set 60 PSI, Ion source gas2 was set 60 PSI, and an interface heater temperature was 500 °C.For positive ion mode, the Ionspray voltage floating were set at 5000 V, respectively. For negative ion mode, the Ionspray voltage floating were set at −4500V, respectively. The mass spectrometry data were acquired in IDA mode.Furthermore, in order to evaluate the stability of the LC-MS during the whole acquisition, a quality control sample (Pool of all samples) was acquired after every 10 samples. The acquired MS data pretreatments including peak picking, peak grouping, retention time correction, second peak grouping, and annotation of isotopes and adducts was performed using XCMS software. LC−MS raw data files were converted into mzXML format and then processed by the XCMS, CAMERA and metaX toolbox implemented with the R software. Each ion was identified by combining retention time (RT) and *m*/*z* data. Intensities of each peaks were recorded and a three dimensional matrix containing arbitrarily assigned peak indices (retention time-m/z pairs), sample names (observations) and ion intensity information (variables) was generated. The raw data were processed for extraction, peak identification, and quality control. Data were sorted by *p*-value, with *p* < 0.05 as the cut-off point, and then the q-value was calculated. q-values <0.05 with a false discovery rate (FDR) of 2.5% were highly statistically significant.

### 4.5 qRT-PCR

Total RNA was extracted from the treated cells using TRIzol reagent (Takara, Japan) according to the manufacturer's protocol. Purified RNA samples were analyzed using a NanoDrop 2000 spectrometer (Thermo Fisher Scientific, USA). cDNA was synthesized using a cDNA synthesis kit (Takara, Japan), and gene expression was assessed using a SYBR Green PCR kit (Thermo Fisher, USA). A Bio-Rad CFX Connect System (Bio-Rad, USA) was used for all qRT-PCR reactions. The relative gene expression was determined using the 2^−ΔΔCT^ method. The specific primers are listed in Additional File 1: Table S1.

### 4.6 Western blotting

Proteins were extracted from the cells with RIPA lysate (Fudebio, China), and protein concentrations were determined using the BCA kit (Abbine, China). Different protein molecules were separated using SDS-PAGE. Proteins were transferred from the gap to a PVDF membrane (Millipore, USA) using the wet transfer method. Non-characterized expression sites were closed using 5% skim milk. The PVDF membranes were co-incubated overnight with the following primary antibodies: GAPDH (1:8000), β-actin (1:10000), ACSS1 (1:1000), E-cadherin (1:5000), N-cadherin (1:2000), vimentin (1:2000), ACLY (1:1000, Proteintech, China), FADS2 (1:500, ABclonal, China), and SCD (1:1000, Cell Signaling Technology, USA). On the second day, the membrane was incubated with a secondary antibody (Proteintech, China) for 1 h at room temperature. Finally, protein signal intensity was detected using enhanced chemiluminescence (ECL, Millipore, USA).

### 4.7 EdU doping assay

EdU-doping experiments were performed as previously described to assess the rate of DNA replication in cells^25^. EdU-positive cells were labeled green. Five fields of view were randomly selected, and the number of positive cells was counted for analysis.

### 4.8 Colony formation assay

To select healthy cells, 1000 cells were inoculated in a six-well plate, and the medium was changed every three days for approximately two weeks. The cells were fixed with 4% paraformaldehyde for 20 min and stained with 0.1% crystal violet.

### 4.9 Scratch healing assay

The cells were inoculated into six-well plates. After reaching 90% cell density, the monolayer was scratched with a 200 μL pipette tip. The scratched cells were washed with PBS. Then, the cells were incubated with serum-free medium and photographed at 0 and 24 h.

### 4.10 Transwell assay

The migration and invasion abilities of the cells were assessed using a transwell assay. Matrigel (BD Biosciences, USA) was added for the invasion assay, and the other two were similar. Briefly, the cell suspension was inoculated into the upper layer of the chamber with serum-free medium, and 700 μL of 10% serum-containing medium was added to the lower layer of the chamber. After 12–24 h of incubation in a constant-temperature incubator, the cells in the lower layer of the chambers were fixed with 4% paraformaldehyde, stained, and counted with 0.1% crystal violet.

### 4.11 3D sphere formation assay

Cells were resuspended in 1X Spheroid Formation ECM (R&D Systems, USA), inoculated into round-bottomed low-adsorption 96-well plates, centrifuged for 3 min at room temperature, and incubated for 48 h. The invasion matrix was added to 96-well plates to continue incubation, and 50 μL of the medium was added every two days. The images were observed under a microscope, and the results were recorded.

### 4.12 Measurement of acetyl-CoA and TG

Acetyl-CoA levels were measured using an acetyl-CoA assay kit (Ruixinbio, China) according to the manufacturer's instructions. The absorbance was measured at 450 nm. The TG content was assessed using a TG assay kit (Nanjing Bio., China) according to the manufacturer's instructions.

### 4.13 Cell immunofluorescence assay

Immunofluorescence was used to localize proteins in cell lines. Briefly, cells were fixed with 4% paraformaldehyde for 30 min. Next, the cells were blocked with 5% bovine serum albumin (BSA) and 0.2% Triton X-100 for 1 h before being incubated overnight at 4 °C with the anti-ACSS1 antibody at a dilution of 1:200 (Proteintech, China) or anti-TOMM20 antibody at a dilution of 1:500 (Huabio, China). The cells were incubated with Alexa Fluor 488-(green) conjugated secondary antibody (Abcam, USA) or Alexa Fluor 647-(pink) conjugated secondary antibody (Bioss, China) at a dilution of 1:200 for 1 h in the dark. The cell nuclei were stained with 4′,6-diamidino-2-phenylindole (DAPI) (blue). The results were visualized using a fluorescence microscope (Nikon, Japan). MitoTracker staining was conducted before subsequent operations using the manufacturer's method (Beyotime, China).

Cells were fixed with 4% paraformaldehyde for 30 min for phalloidin staining and co-stained with 100 nM working solution for 20 min. The results were observed and recorded using a fluorescence microscope.

### 4.14 BODIPY 493/503 and C11-BODIPY staining

The lipid droplets were stained with the Bodipy 493/503 probe. The cells were cultured on glass coverslips, fixed, washed, and incubated with 1 μM Bodipy 493/503 for 30 min at room temperature. Then, nuclei were stained with Hoechst 33342 (Keygen Bio., China), observed, and photographed under a fluorescence microscope.

Lipid peroxidation was measured using C11-BODIPY. When lipid peroxidation increased, the intensity of red fluorescence decreased. C11 BODIPY 581/591 (Abclonal, China) staining was conducted according to the manufacturer's protocol.

### 4.15 Measurement of ROS

The DCFH-DA probe was used to detect intracellular ROS levels. The working solution was prepared at a ratio of 1:1000, and the cells were washed with PBS. The cells were co-incubated with the working solution for 30 min before the data were recorded using CytoFLEX and analyzed using FlowJo 10.8.1.

### 4.16 TEM

The treated cells were fixed with a 2.5% glutaraldehyde solution at 4 °C for 4 h. The samples were sequentially dehydrated, embedded, cured, sectioned, and double-stained. Finally, the cells were observed and photographed using a transmission electron microscope (HITACHI, Japan).

### 4.17 Seahorse analysis

A mitochondrial stress assay kit was used to assay mitochondrial respiration rates under basal conditions in the presence of oligomycin, FCCP, antimycin A, and rotenone. The OCR was measured using a Seahorse XFe96 analyzer (Agilent, USA) following the manufacturer's protocol.

### 4.18 Mouse tumor xenografts

All animal experiments were approved by the Ethics Committee of the ∗∗∗∗∗∗∗University (No.∗∗∗∗∗∗∗). The nude mice resided in the SPF-grade laboratory animal center. Cells transfected with the luciferase plasmid were inoculated into the bone marrow cavity of the tibia of nude mice at one million cells each. When the tumor size approached the ethical limit, the tumor tissue was removed, and subsequent experiments were performed. For the metastatic model, cells were injected into the blood system of nude mice via the tail vein and observed for lung colonization.

### 4.19 H&E

H&E staining was performed as described previously [26].

### 4.20 Immunohistochemistry (IHC) analysis

The tumor tissues were sectioned after fixation and paraffin embedding. After routine dewaxing and hydration, the specimens were repaired for antigens and closed with BSA, followed by incubation with anti-ACSS1 (1:200), anti-vimentin (1:200), anti-N-cadherin (1:200), or anti-Ki67 (1:5000) (Proteintech, China). Secondary staining was performed with HRP-conjugated anti-rabbit IgG and DAB peroxidase substrate. Finally, the results were recorded under a microscope after sealing them with neutral gum. The IHC scores for ACSS1 were evaluated independently by two pathologists. Staining was quantified using a semi-quantitative method based on the percentage of positive cells and staining intensity. The percentage of positive cells was scored as follows: 1 (0–25%), 2 (26–50%), 3 (51–75%), and 4 (76–100%). Staining intensity was graded on a scale of 0 to 3. A final IHC score for each sample was calculated by multiplying the positive-cell percentage score by the intensity score. Patients were then stratified into two groups based on these IHC scores for overall survival analysis, with a p-value <0.05 considered statistically significant.

### 4.21 Bioinformatic and statistical analysis

Data from https://www.ncbi.nlm.nih.gov/geo/. Gene set enrichment analysis (GSEA) was performed using GSEA software (https://www.gsea-msigdb.org/gsea/). All data statistics were analyzed using GraphPad Prism9.0 or SPSS22.0. Categorical data were analyzed using Pearson's chi-square or Fisher's test. Survival data were analyzed using the log-rank test. Correlations were analyzed using Pearson's correlation analysis. The two groups were compared using the t-test or the Mann–Whitney U-test. Multiple groups were compared using a one-way ANOVA or Tukey's test. The data were expressed as mean ± standard deviation (SD). The statistical analysis threshold was set at *p* < 0.05 for significant differences.

## 5 Contributions

ZZ, ZS and LL conceived the idea of the project. XH, ML, CW and LK performed the experiments. JL, JT collected and analyzed subject data. JC, YN and HC contributed to the analysis and visualization of data. XH, ML, CW and LK wrote and edited the manuscript. ZZ, ZS and LL supervised the study, acquired funding, and oversaw the project. All authors reviewed and edited the manuscript.

## Ethics declarations

This study was approved by the Ethics Committee of Zhujiang Hospital, Southern Medical University (Guangzhou, China) (human OS study: 2018-GJGBWK-002; animal experimental procedures: LAEC- 2022-043). All experiments were performed in accordance with the Declaration of Helsinki. Patients with OSA who participated in this study provided informed consent.

## Funding

This research was supported by 10.13039/501100001809National Natural Science Foundation of China (Grant no. 82572792 and 82503822), 10.13039/501100003453Natural Science Foundation of Guangdong Province (Grant no.2022A1515010293), Presidential Foundation of Zhujiang Hospital (Project No.yzjj2022ms15 and yzjj2023qn25), and Zhongshan City Social Welfare and Basic Research Project (Medical and Health) (No. 231227135028664).

## Declaration of Generative AI in scientific writing statement

No generative artificial intelligence (AI) or AI-assisted technologies were used in the preparation of this manuscript.

## Conflict of interest

The authors state that they have no identifiable financial conflicts or personal affiliations that may have impacted the findings reported in this paper.

## Figures and Tables

**figs1:**
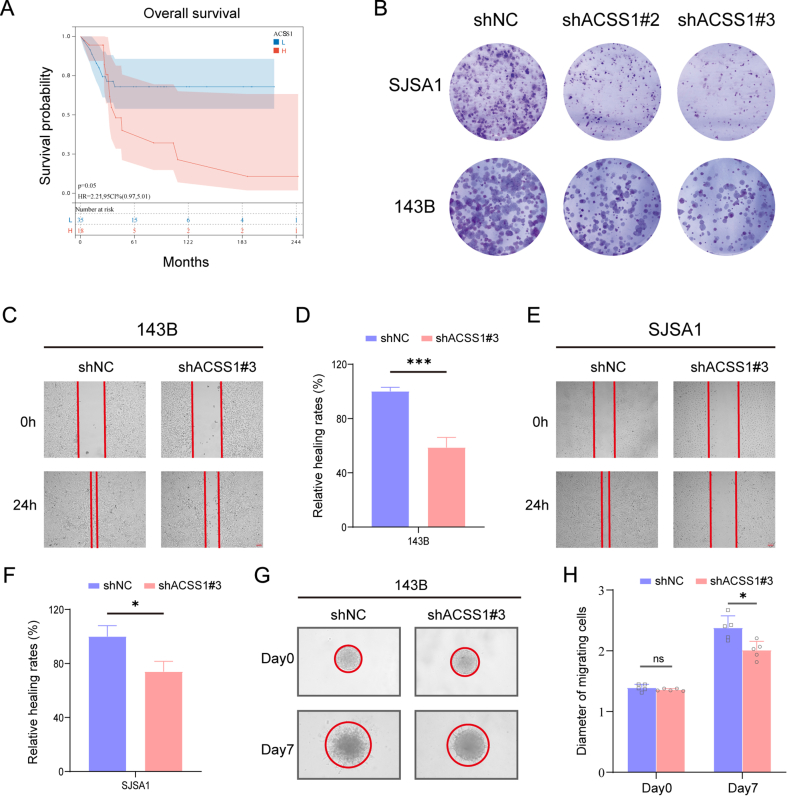


**figs2:**
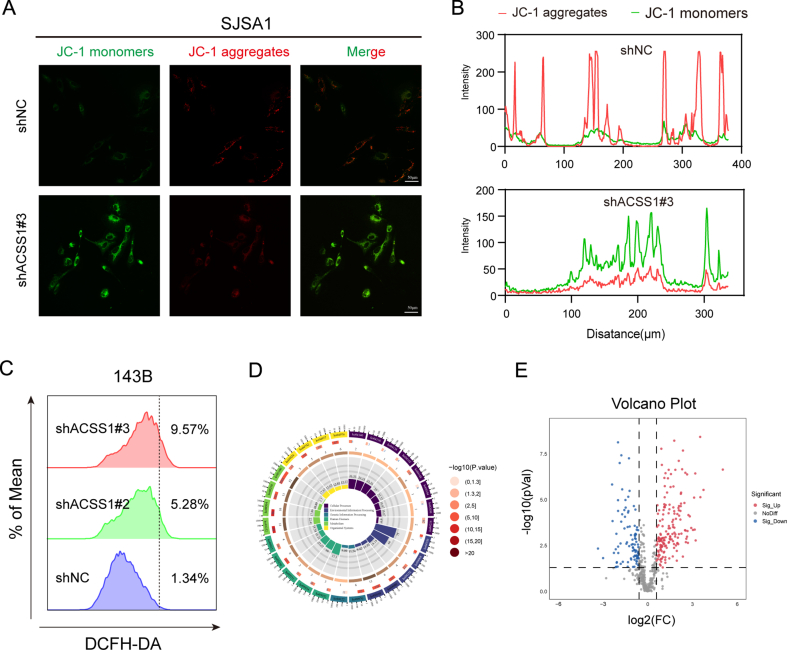


**figs3:**
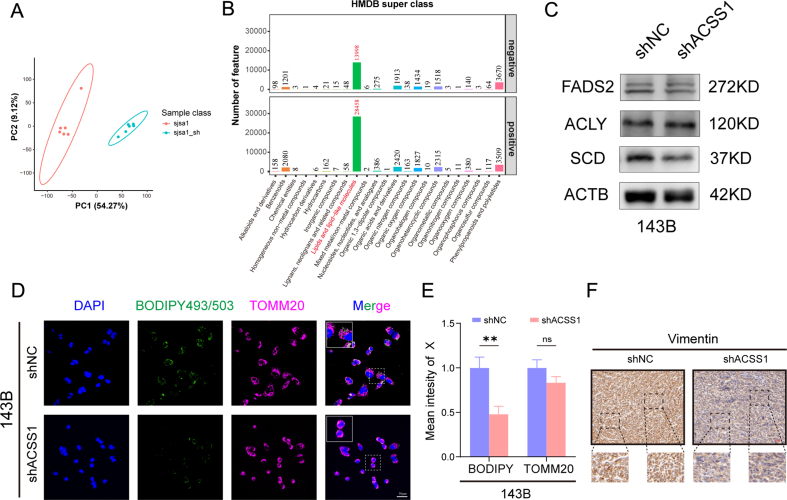


**figs4:**
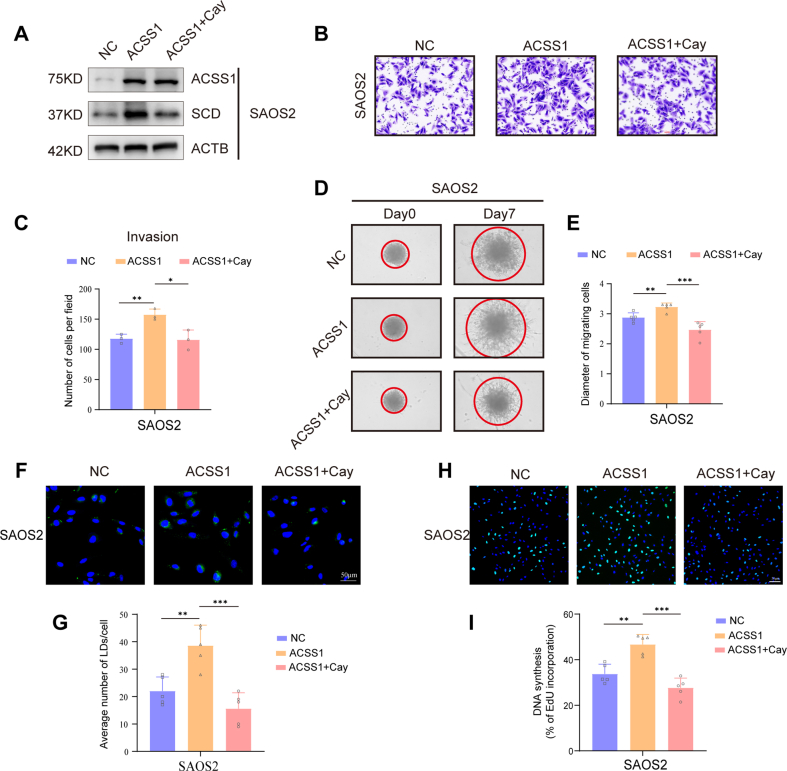


**figs5:**
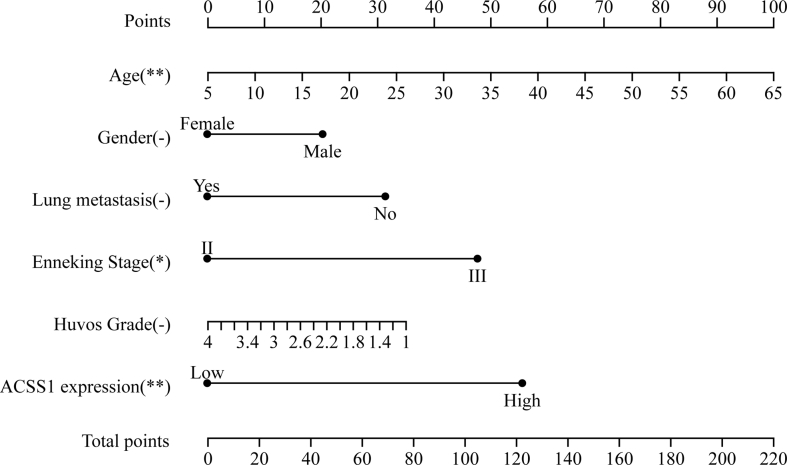


## Data Availability

The data that support the findings of this study are available from the corresponding authors on request.
