# Supplementary material for: Acetyl-CoA synthase 1 mediates metabolic reprogramming to promote proliferation and metastasis of osteosarcoma
Source: J Orthop Translat. 2026 Apr 1;57:101052. doi: 10.1016/j.jot.2026.101052 (PMC13081702; doi:10.1016/j.jot.2026.101052)
Supplement: Multimedia component 1 [file mmc1.docx]

**Fig. 1**

**
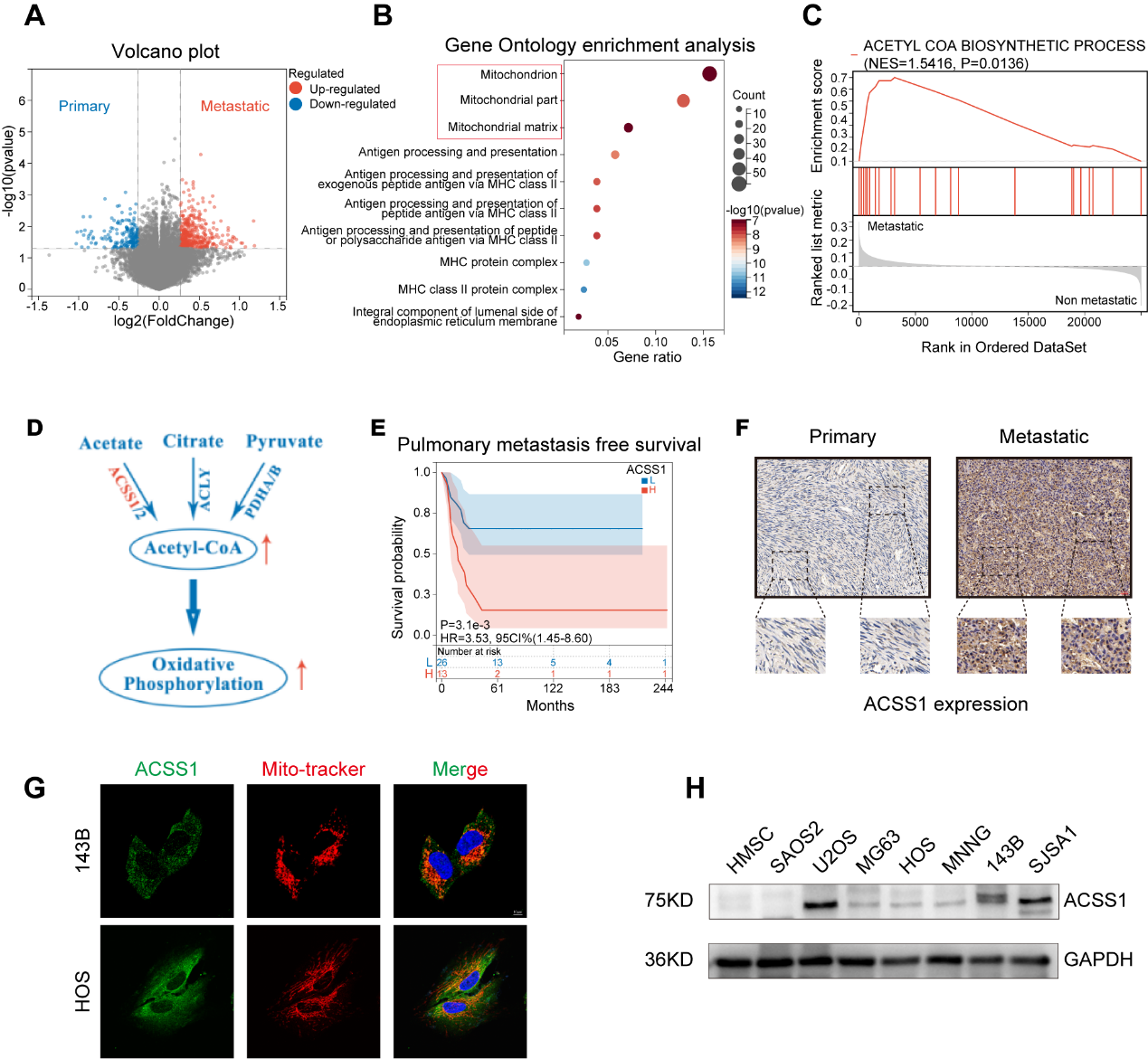
**

**A** Volcano plots showing the differential genes in metastatic versus non-metastatic OS tissues in GSE21257. **B** GO functional enrichment analysis of significantly differential genes. **C** The acetyl coenzyme A biosynthetic process in GSEA analysis. **D** Pattern diagram of acetyl coenzyme A biosynthetic process. **E** Pulmonary metastasis-free survival analysis of different ACSS1 expression in OS patients. **F** Immunohistochemical analysis of ACSS1 expression in specimens from patients with OS. **G** Immunofluorescence analysis showing the localization of ACSS1 in OS cells. **H** Protein expression of ACSS1 in OS cell lines and HMSC.

**Fig. 2**


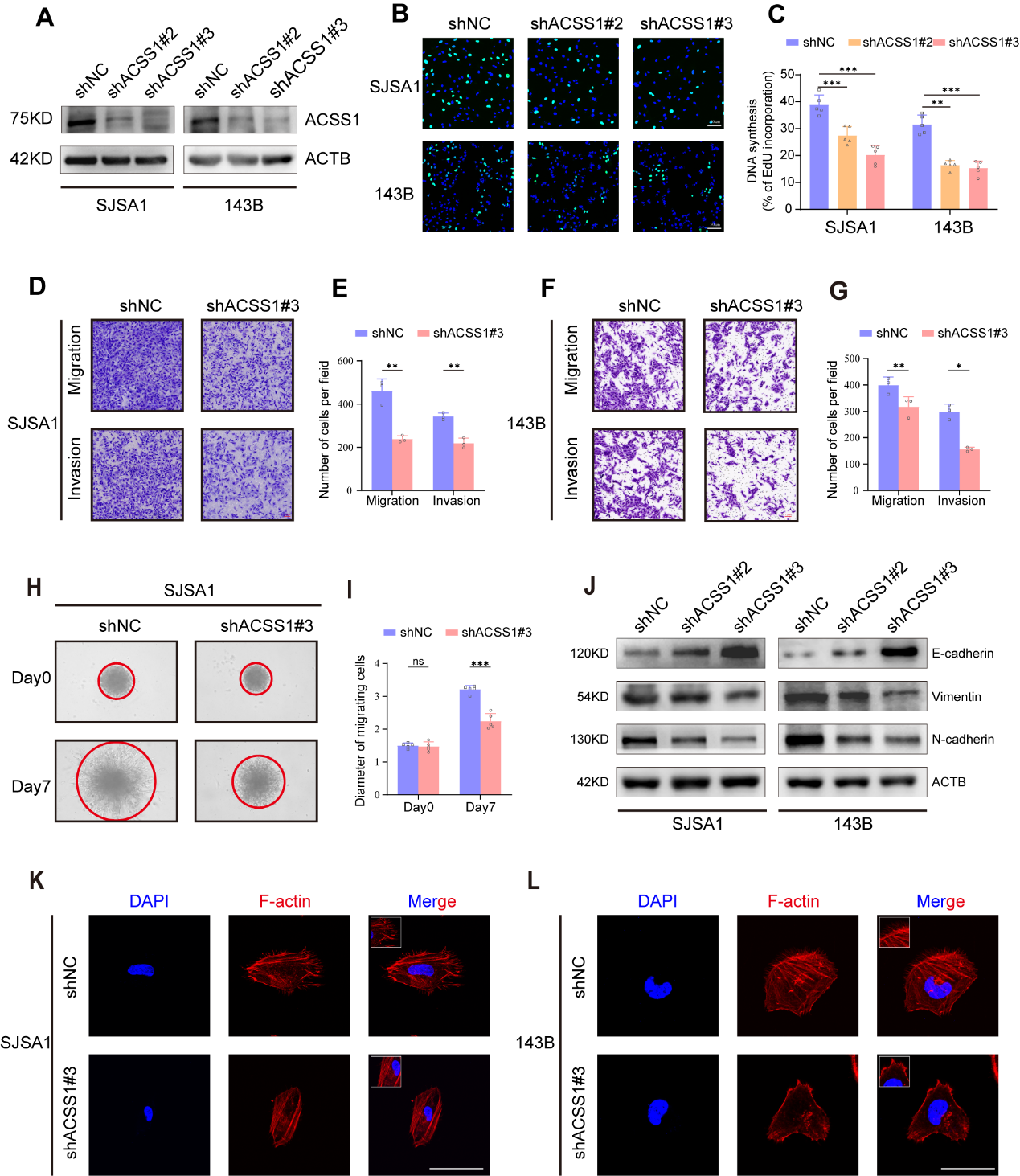


**A** Western blotting detection of the protein knockdown efficiency of ACSS1 in SJSA1 and 143B cells. **B** EdU doping assay showing the DNA replication capacity of SJSA1 and 143B cells. **C** For the statistical graph in b, five random captures were taken for statistical purposes. **D-E** Migration and invasion ability of SJSA1 cells detected using Transwell assay. **F-G** Migration and invasion ability of 143B cells detected using Transwell assay. **H** 3D sphere-forming assay showing the invasive ability of SJSA1 cells. **I** The statistics are plotted in terms of the diameter of the infiltrating cells based on h. **J** Western blotting was used to detect vimentin, N-cadherin, and E-cadherin protein expression levels. **K-I** Phalloidin staining detecting the number of pseudopods on the surface of OS cells after ACSS1 knockdown. Scale bar: 100 µm. Data are expressed as the mean ± SD. **p* < 0.05; ***p* < 0.01; ****p* < 0.001; ns. not significant.

**Fig. 3**


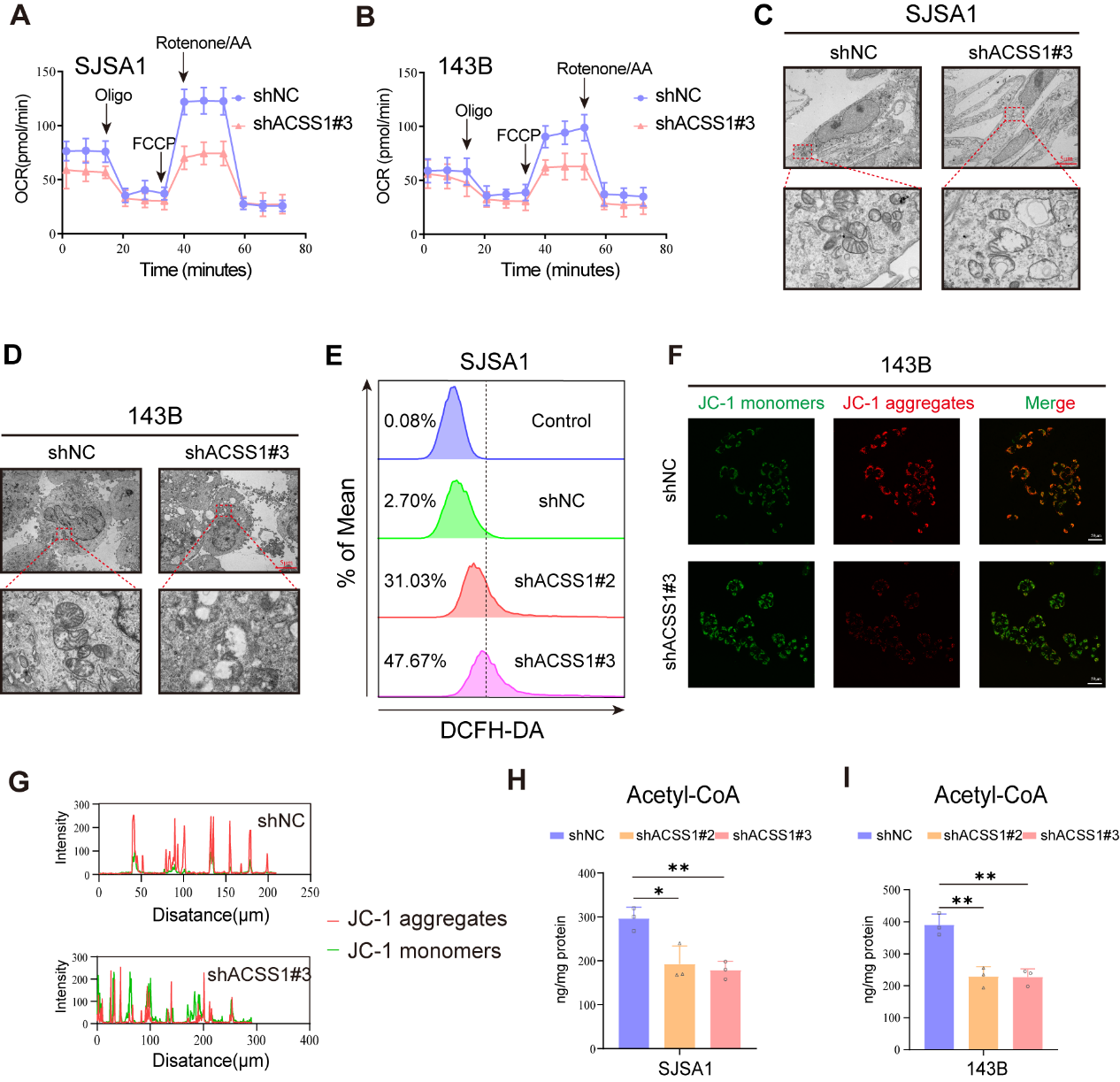


**A-B** Seahorse assay to detect intracellular oxygen consumption and mitochondrial reserve capacity in OS cells after ACSS1 knockdown. **C-D** Transmission electron microscopy of mitochondrial structure in OS cells after reduced ACSS1 expression. **E** Intracellular reactive oxygen species levels in SJSA1 cells under ACSS1 knockdown were detected using flow cytometry. **F-G** Staining of mitochondrial JC-1 membrane potential and fluorescence intensity graphs in 143B cells after ACSS1 knockdown. **H-I** Alterations in acetyl coenzyme A in OS cells after ACSS1 knockdown were detected using ELISA. Data are expressed as the mean ± SD. **p* < 0.05; ***p* < 0.01; ****p* < 0.001; ns. not significant.

**Fig. 4**


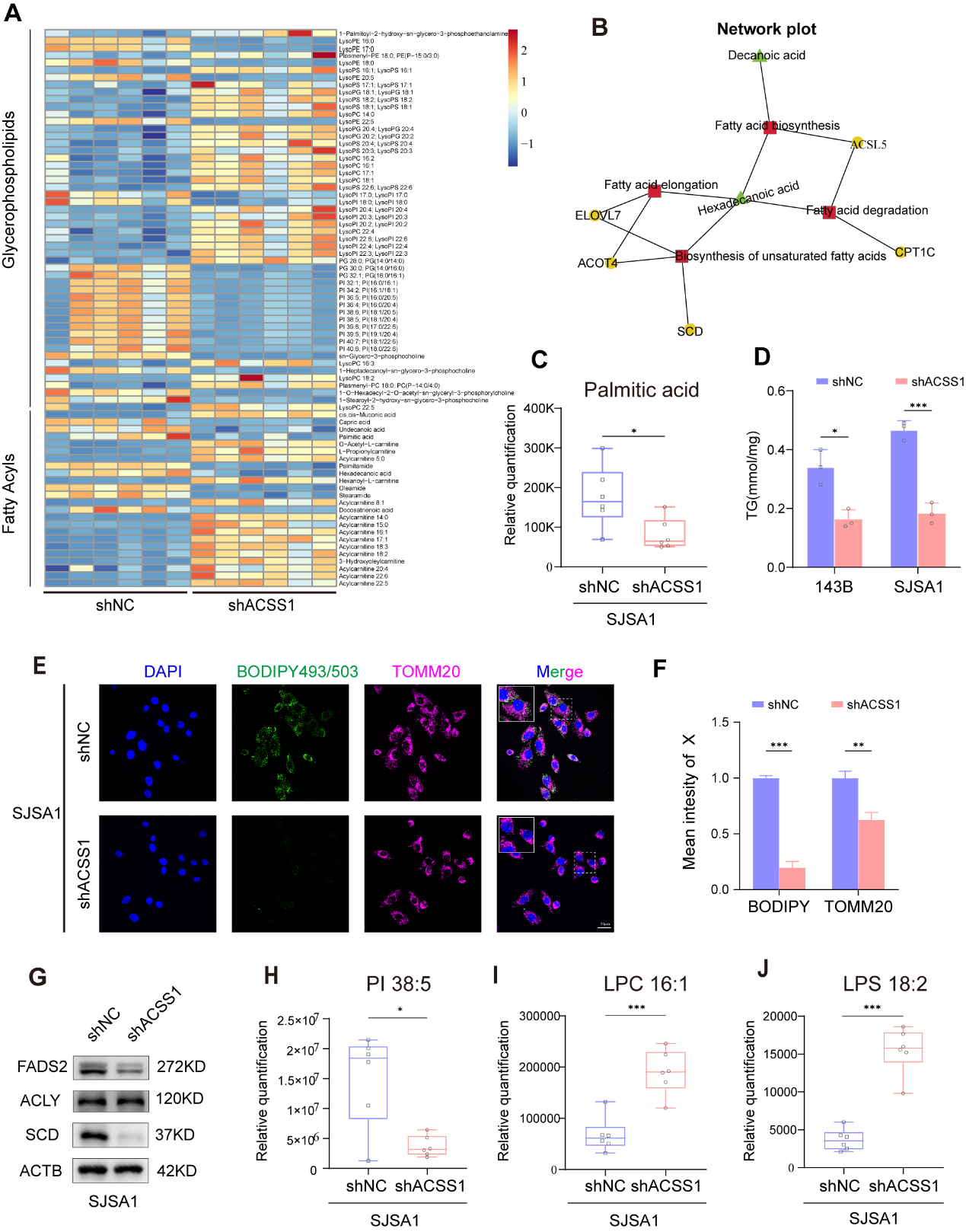


**A** Heatmap of representative metabolite species in ACSS1-knockdown SJSA1 cells (n = 6) versus control cells (n = 6) analyzed using untargeted metabolomics. **B** A net plot of the combined transcriptomics and untargeted metabolomic analysis of ACSS1 knockdown in SJSA1 cells. **C** Palmitate metabolite content in untargeted metabolomics after ACSS1 knockdown. **D** Determination of TG content after ACSS1 knockdown. **E-F** Detection of lipid droplet content and TOMM20 expression by immunofluorescence in SJSA1 cells. **G** Western blotting detection of fatty acid metabolism-related gene changes in SJSA1 cells. **H–J** Expression of PI, LPC, and LPS in untargeted metabolomics. Data are expressed as the mean ± SD. **p* < 0.05; ***p* < 0.01; ****p* < 0.001; ns. not significant.

**Fig. 5**


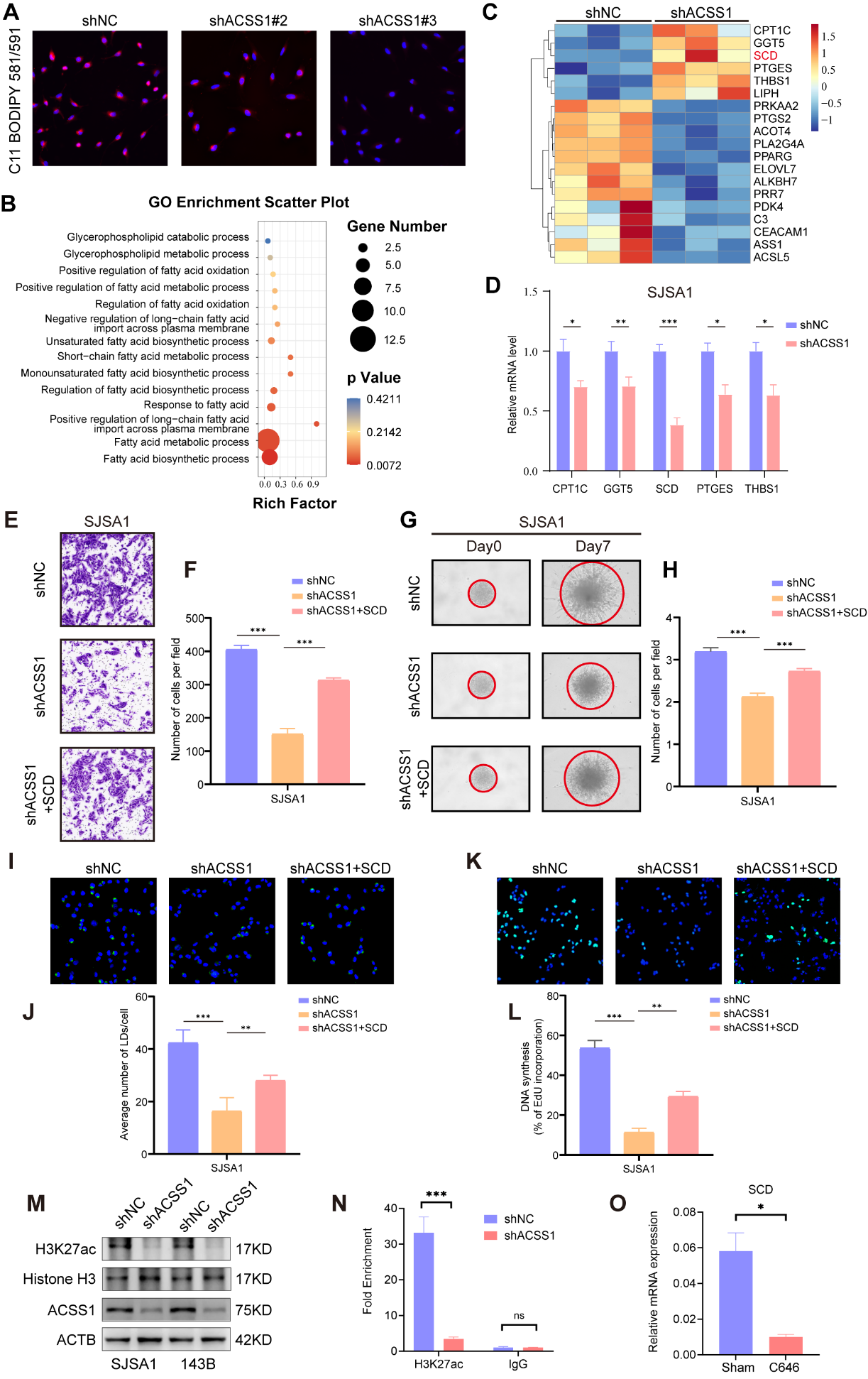


**A** C11 BODIPY581/591 assay for lipid peroxidation levels after ACSS1 knockdown in SJSA1 cells. **B** Heatmap of transcriptomics of knockdown ACSS1 (n = 3). **C** GO analysis was conducted to look for biological processes after ACSS1 knockdown. **D** Relative mRNA expression of the five candidate downstream genes was determined using qRT-PCR. **E-F** Transwell assay to detect the effect of ACSS1/SCD axis on OS cell invasion. **G-H** 3D sphere-forming assay to detect the effect of the ACSS1/SCD axis on cell tumorigenesis. **I-J** Effect of ACSS1/SCD axis on lipid droplet content detected by BODIPY 493/503 staining. **K-L** EdU doping assay to detect the effect of the ACSS1/SCD axis on cell proliferation. **M** Western blotting detection of acetylation H3K27 in osteosarcoma cells. **N** ChIP‐qPCR analysis showing reduced H3K27ac enrichment after ACSS1 knockdown. **O** Relative mRNA expression of SCD after inhibition of P300 with C646. Data are expressed as the mean ± SD. **p* < 0.05; ***p* < 0.01; ****p* < 0.001; ns. not significant.

**Fig. 6**


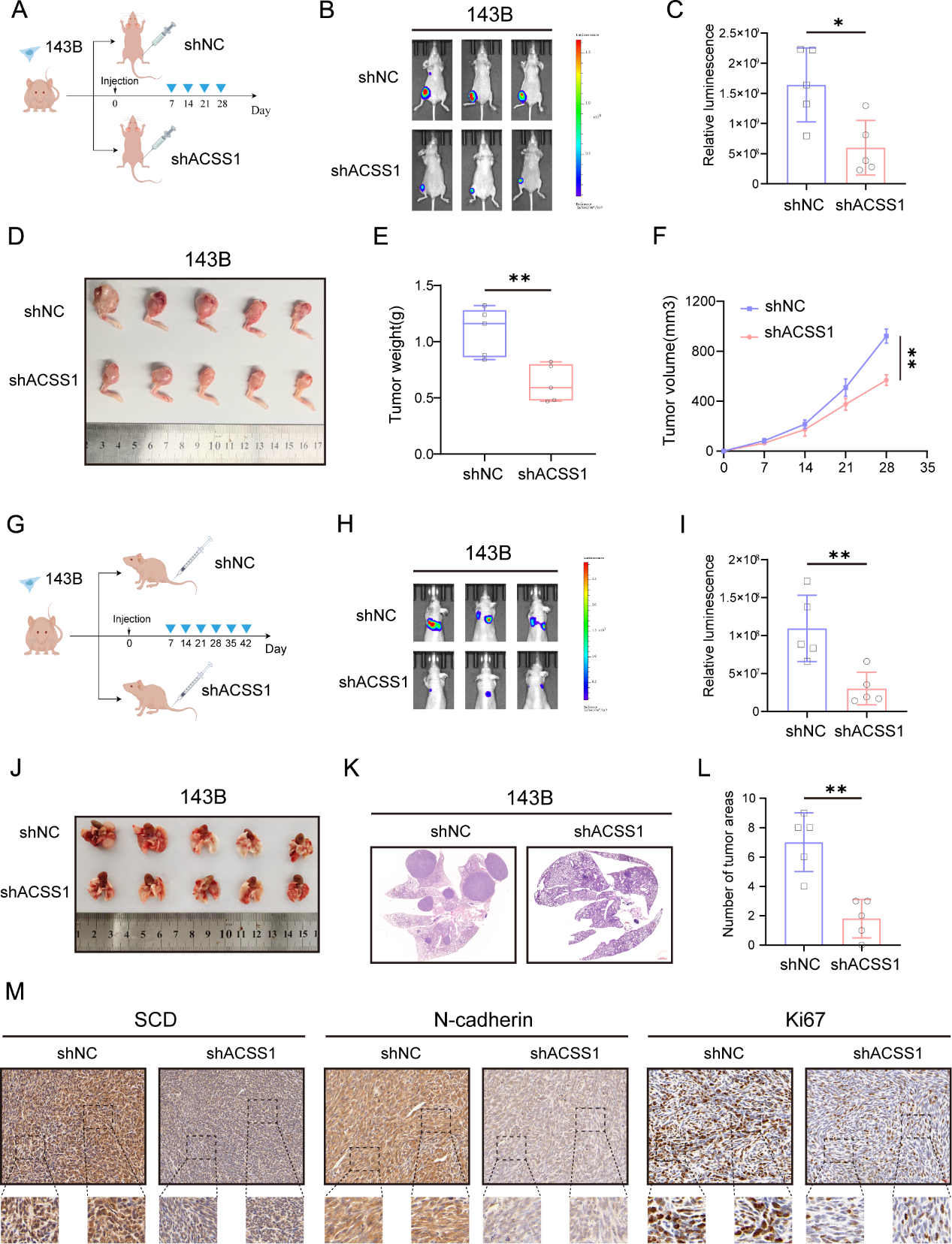


**A** Diagram of the orthotopic xenograft model of the right tibia in nude mice (n=5). **B-C** A small animal *in vivo* optical imaging system was used to observe the fluorescence intensity values between knockdown ACSS1 and controls. **D–F** Tumor bulk, weight, and volume between the knockdown ACSS1 and control groups. **G** Diagram of the nude mouse tail vein lung metastasis xenograft model (n = 5). **H-I** The effects of the ACSS1 knockdown on lung metastasis formation were visualized by live imaging in small animals. **J** A gross view of the metastases in the lungs. **K-L** H&E staining was utilized to assess the pathological findings in the lungs. **M** Immunohistochemical staining was performed to observe the effect of ACSS1 on the expression of SCD, N-cadherin, and Ki67. Data are expressed as the mean ± SD. **p* < 0.05; ***p* < 0.01; ****p* < 0.001; ns. not significant.

**Fig. 7**


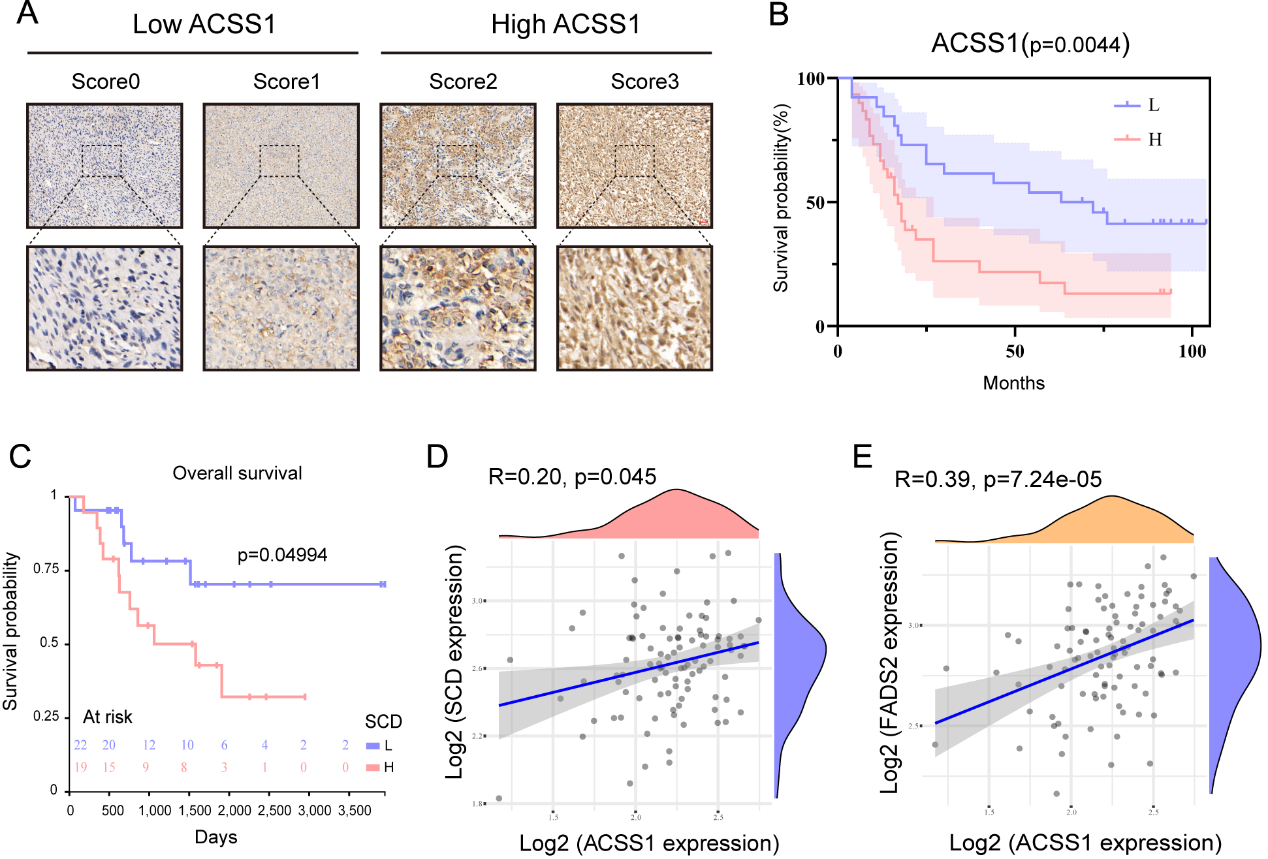


**A** Immunohistochemical scores distinguish high and low ACSS1 expression in tissue samples. **B** Analysis of ACSS1 expression in tissues and overall survival of OS patients. **C** Prognostic analysis of SCD and overall survival in patients with OS. **D** Correlation analysis between ACSS1 and SCD. **E** Correlation analysis between ACSS1 and FADS2.

**Fig. S1**


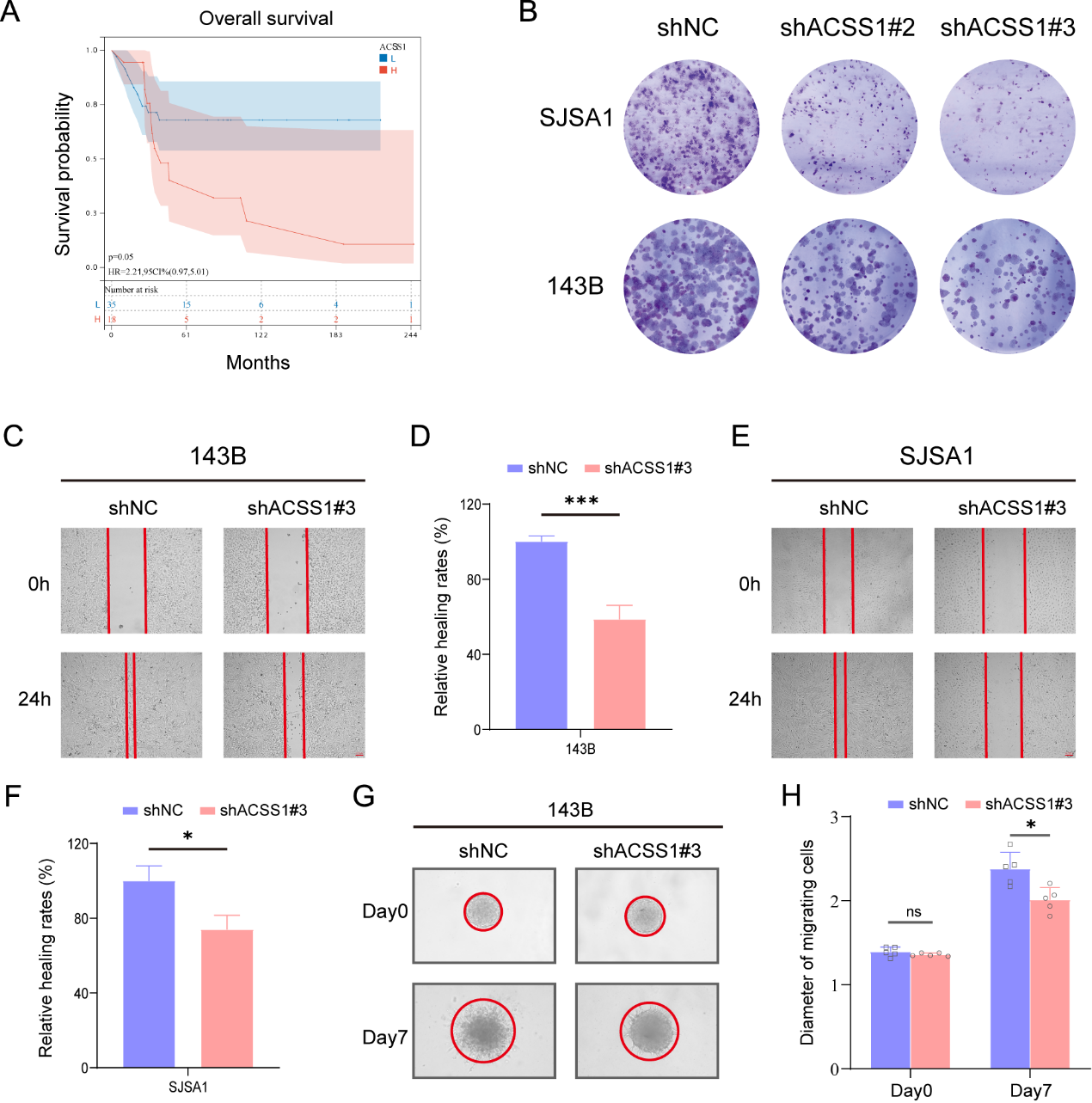


**A** Prognostic analysis of ACSS1 expression and overall survival in patients with OS. **B** Clone formation assays were performed to detect the proliferative capacity of SJSA1 and 143B cells. **C–F** Migration ability of OS cells detected by scratch healing assay. **G-H** 3D sphere-forming assay to detect the invasive ability of 143B cells. Data are expressed as the mean ± SD. **p* < 0.05; ***p* < 0.01; ****p* < 0.001; ns. not significant.

**Fig. S2**


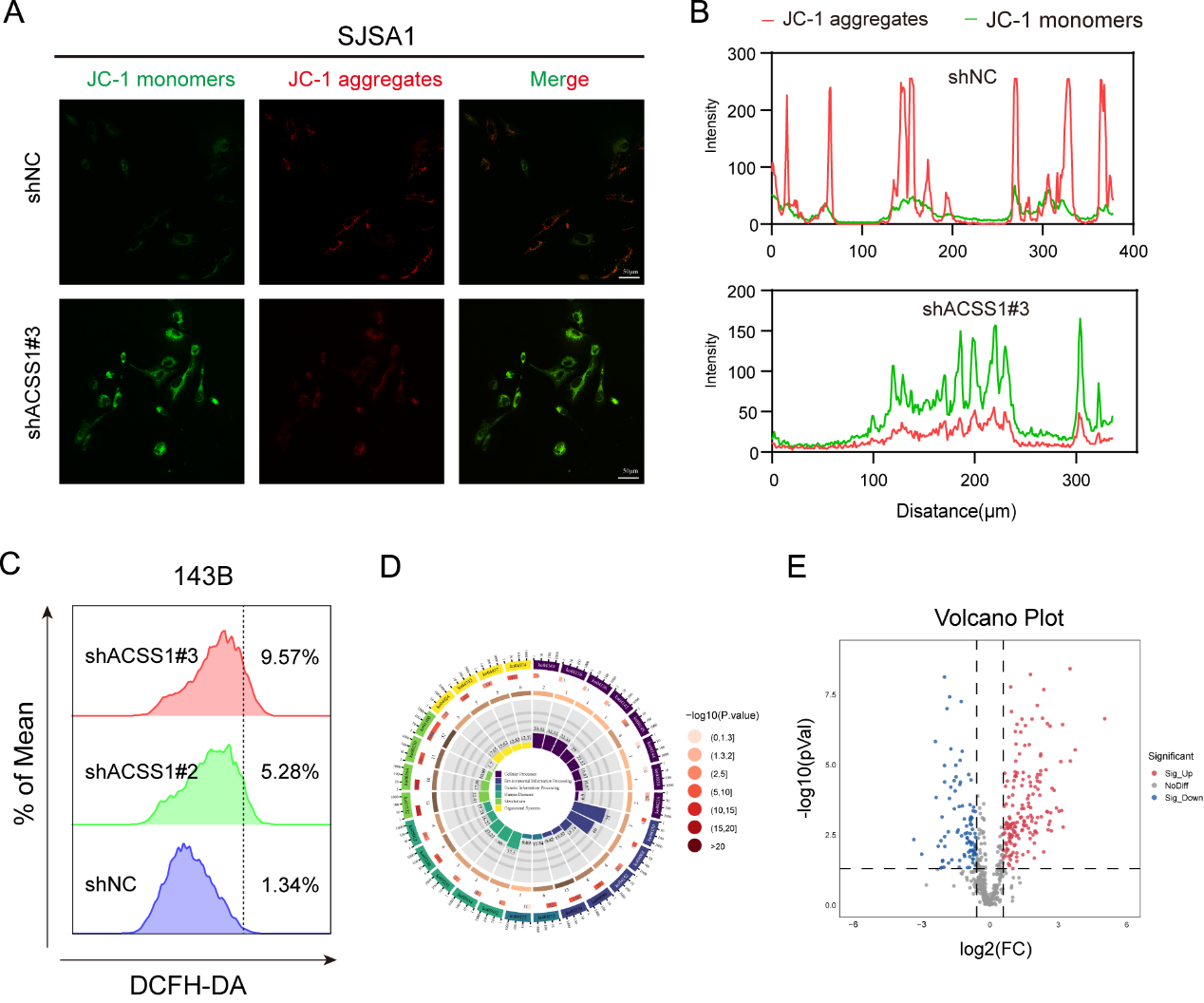


**A-B** Staining of mitochondrial JC-1 membrane potential and fluorescence intensity graphs in SJSA1 cells after ACSS1 knockdown. **C** Intracellular reactive oxygen species levels in 143B cells under ACSS1 knockdown were detected using flow cytometry. **D** KEGG enrichment analysis of differential metabolites. **E** Volcano plot of differential metabolites between ACSS1 knockdown and controls.

**Fig. S3**


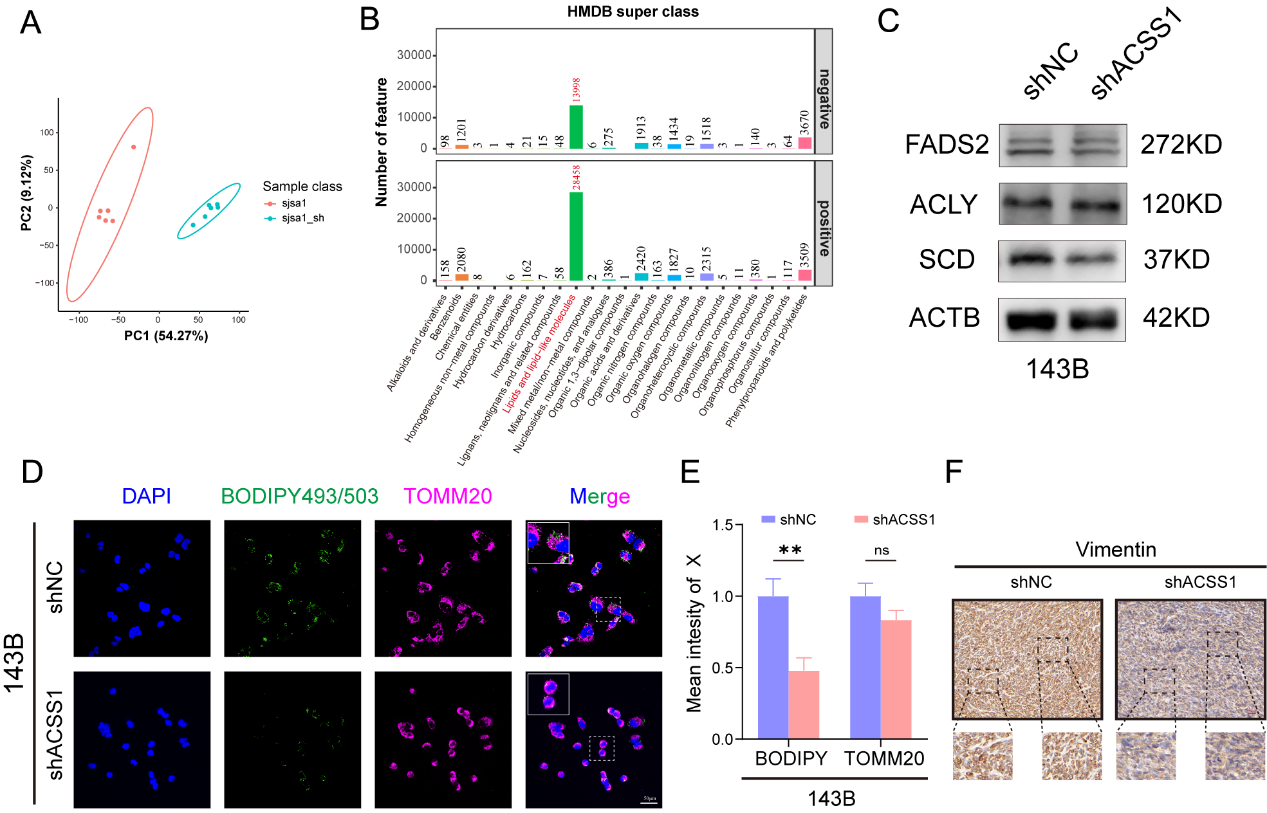


**A** PCA component analysis in untargeted metabolomics between knockdown ACSS1 and controls. **B** Metabolite distribution in untargeted metabolomics. **C** Western blotting detection of fatty acid metabolism-related gene changes in 143B cells. **D-E** Detection of lipid droplet content and TOMM20 expression using immunofluorescence in 143B cells. Data are expressed as the mean ± SD. **p* < 0.05; ***p* < 0.01; ****p* < 0.001; ns. not significant. **F** Immunohistochemical staining was performed to observe the effect of ACSS1 on the expression of Vimentin. Data are expressed as the mean ± SD. **p* < 0.05; ***p* < 0.01; ****p* < 0.001; ns. not significant.

**Fig. S4**


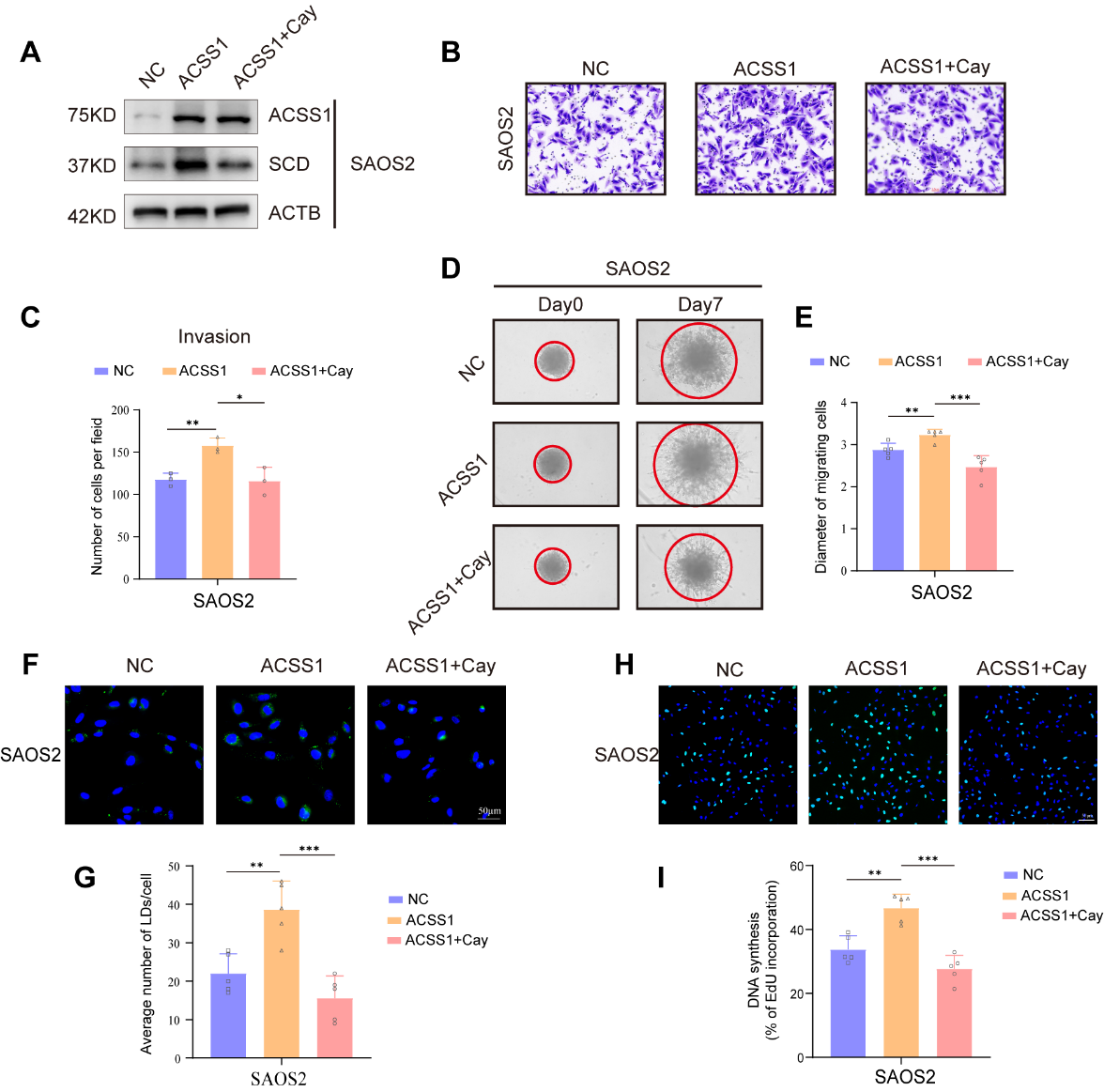


**A** Western blotting to detect the expression of SCD and ACSS1 after overexpression of ACSS1 and with the concomitant addition of SCD inhibitor Cay in SAOS2 cells. **B-C** Transwell assay to detect the effect of ACSS1/SCD axis on OS cell invasion. **D-E** 3D sphere-forming assay to detect the effect of the ACSS1/SCD axis on cell tumorigenesis. **F-G** Effect of ACSS1/SCD axis on lipid droplet content detected by BODIPY 493/503 staining. **H-I** EdU doping assay to detect the effect of the ACSS1/SCD axis on cell proliferation. Data are expressed as the mean ± SD. **p* < 0.05; ***p* < 0.01; ****p* < 0.001; ns. not significant.

**Fig. S5**


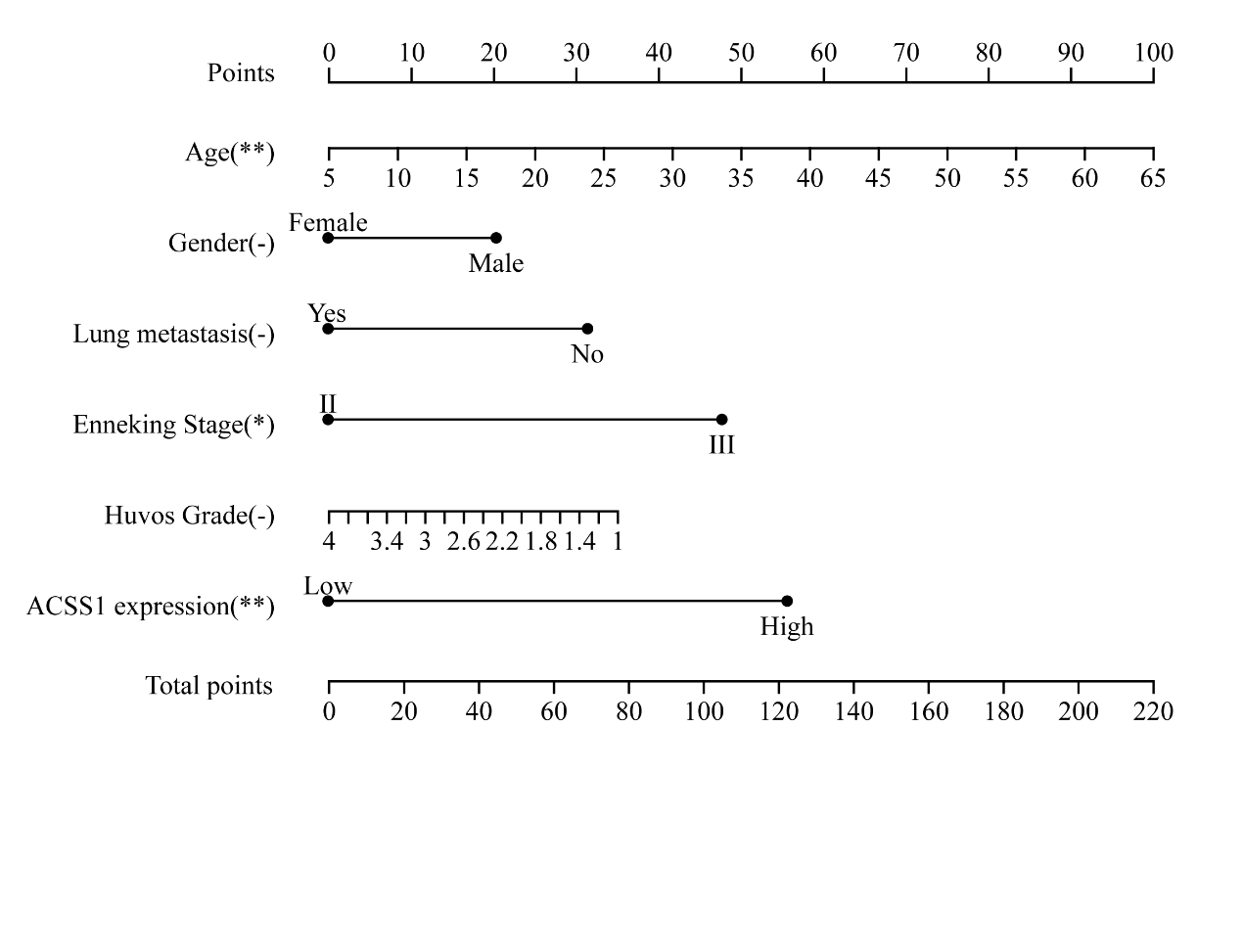


Multivariate regression analysis suggests that ACSS1 can serve as a predictive indicator for overall survival prognosis in patients with osteosarcoma.

**Table1:** Clinical characteristics of 56 patients with OS.

| **Clinicopathological features** | **Relative ACSS1 expression** | | ***P*-value**  **(χ^2^ test)** |
| --- | --- | --- | --- |
|  | **Low(n=26)** | **High(n=30)** |  |
| *Gender* |  |  | 0.333 |
| male | 18 | 17 |  |
| female | 8 | 13 |  |
| *Age* |  |  | 0.136 |
| ≤18 | 4 | 11 |  |
| >18 | 22 | 19 |  |
| *Distant metastasis* |  |  | 0.035* |
| *Absent* | 16 | 10 |  |
| *Present* | 10 | 20 |  |

**TableS1:** Primer sequences for qRT-PCR in this study

| Gene | Forward primer (5’ to 3’) | Reverse primer (5’ to 3’) |
| --- | --- | --- |
| CPT1C | TCAGAGGGTCAGGGAAGGAGAAC | GGAGTCAGAAGTCGGTGGATGTC |
| GGT5 | AGATGCTGGTGGAGGACATTGC | GCGGCGGTGGTGAGTACAG |
| SCD | CGTCCGTGTGTCCCAGATGC | GGCTAGTTATCCACCGCTTCTCC |
| PTGES | CCCGCTGACGCTTCCCTTG | CCTCCACCCACTGCCCTTTG |
| THBS1 | GGGTTTCCTCCTTCTGGCATCC | GGACACCACGCTGAAGACCTG |
| GAPDH | CTCCAAAATCAAGTGGGGCG | TGGTTCACACCCATGACGAA |
